# Differential methylation of enhancer at *IGF2* is associated with abnormal dopamine synthesis in major psychosis

**DOI:** 10.1038/s41467-019-09786-7

**Published:** 2019-05-03

**Authors:** Shraddha Pai, Peipei Li, Bryan Killinger, Lee Marshall, Peixin Jia, Ji Liao, Arturas Petronis, Piroska E. Szabó, Viviane Labrie

**Affiliations:** 1 The Donnelly Centre, University of Toronto, Toronto, M5S 3E1 ON Canada; 2 The Centre for Addiction and Mental Health, Toronto, M5T 1R8 ON Canada; 3 Center for Neurodegenerative Science, Van Andel Research Institute, Grand Rapids, 49503 MI USA; 4 Krembil Family Epigenetics Laboratory, Centre for Addiction and Mental Health, Toronto, M5T 1R8 ON Canada; 5 Center for Epigenetics, Van Andel Research Institute, Grand Rapids, 49503 MI USA; 6 Institute of Biotechnology, Life Sciences Center, Vilnius University, LT-10257 Vilnius, Lithuania; 7 Division of Psychiatry and Behavioral Medicine, College of Human Medicine, Michigan State University, Grand Rapids, 49503 MI USA

**Keywords:** Epigenomics, Gene regulation, Chromatin structure, Psychosis, Genetics of the nervous system

## Abstract

Impaired neuronal processes, including dopamine imbalance, are central to the pathogenesis of major psychosis, but the molecular origins are unclear. Here we perform a multi-omics study of neurons isolated from the prefrontal cortex in schizophrenia and bipolar disorder (n = 55 cases and 27 controls). DNA methylation, transcriptomic, and genetic-epigenetic interactions in major psychosis converged on pathways of neurodevelopment, synaptic activity, and immune functions. We observe prominent hypomethylation of an enhancer within the insulin-like growth factor 2 (*IGF2*) gene in major psychosis neurons. Chromatin conformation analysis revealed that this enhancer targets the nearby tyrosine hydroxylase (*TH*) gene responsible for dopamine synthesis. In patients, we find hypomethylation of the *IGF2* enhancer is associated with increased TH protein levels. In mice, *Igf2* enhancer deletion disrupts the levels of TH protein and striatal dopamine, and induces transcriptional and proteomic abnormalities affecting neuronal structure and signaling. Our data suggests that epigenetic activation of the enhancer at *IGF2* may enhance dopamine synthesis associated with major psychosis.

## Abstract (web summary)

Dopamine dysregulation is centrally linked to major psychosis. Here, the authors characterise the hypomethylation of an enhancer within the insulin-like growth factor 2 gene in neurons of patients with major psychosis and provide evidence that this enhancer targets the tyrosine hydroxylase gene, responsible for dopamine synthesis.

## Introduction

Schizophrenia and bipolar disorder are mental disorders characterized by periods of psychosis, including hallucinations, delusions, and thought disorder. These diseases have shared genetic features, peri-adolescent onset, and dynamic clinical symptoms, and affect 100 million people worldwide^1^. Psychotic symptoms are thought to be triggered by dopaminergic dysregulation, as the efficacy of all actively used antipsychotic drugs involves an attenuation of dopamine transmission, and the dopamine hypothesis of schizophrenia has endured as a neurochemical explanation for disease pathogenesis for over 60 years^2^. In addition, neurons of patients with psychosis exhibit numerous transcriptional, structural (decreases in dendritic spine density), and signaling abnormalities that disrupt cortical circuitry^3–7^. The past decade of genomics research has shown that epigenetic misregulation of the genome can trigger long-lasting changes to neurodevelopmental programs, synaptic architecture, and cellular signaling, and thus may increase the risk of psychotic disorders, such as schizophrenia and bipolar disorder^5,8–10^. In particular, abnormalities in DNA methylation have been detected in the brain of schizophrenia and bipolar disorder patients, and their involvement in disease pathophysiology could explain the clinical dynamics observed in these diseases^11–13^. However, DNA methylation studies of bulk brain tissue are confounded by sample-level variation in the proportion of different cell types. In addition, epigenetic changes occurring within neurons can be masked by the predominant glial signal; there are ~3.6 times more glia than neurons in the human frontal cortex gray matter^14^. Epigenomic profiling in neurons of affected individuals – rather than blood or cell mixtures – would provide more accurate data for a model of neuronal dysregulation in disease; to date, no such data are available.

In this work, we perform a genome-wide comparison of DNA methylation in isolated neurons from the frontal cortex of individuals with schizophrenia and bipolar disorder, to those in undiagnosed individuals. We report a strong association in an enhancer located within the *IGF2* locus, using an array-based approach, and by targeted bisulfite deep sequencing. *IGF2* has been previously been found to be differentially methylated in populations at risk for schizophrenia^15^, and affects synaptic plasticity and cognitive functions like learning and memory^16–20^. We then use several functional assays, bioinformatics, and mouse transgenics to provide evidence that the enhancer at *IGF2* regulates the tyrosine hydroxylase (*TH*) gene; TH is the rate-limiting enzyme responsible for dopamine synthesis. We also find that *Igf2* enhancer disruption in mice affects levels of TH protein and dopamine, as well as pathways involved in synaptic signaling and neuronal structure. This work suggests a mechanism for epigenetic regulation of dopamine levels in the brain. Epigenetic misregulation of an enhancer at *IGF2* may underlie the dopaminergic abnormalities that drives psychotic symptoms. The epigenetic regulatory connection between *IGF2* and *TH* may also help explain the co-occurrence of neuronal structure and synaptic abnormalities with dopamine dysregulation in major psychosis patients^21–23^.

## Results

### DNA methylome abnormalities in psychosis patient neurons

**Fig. 1:**
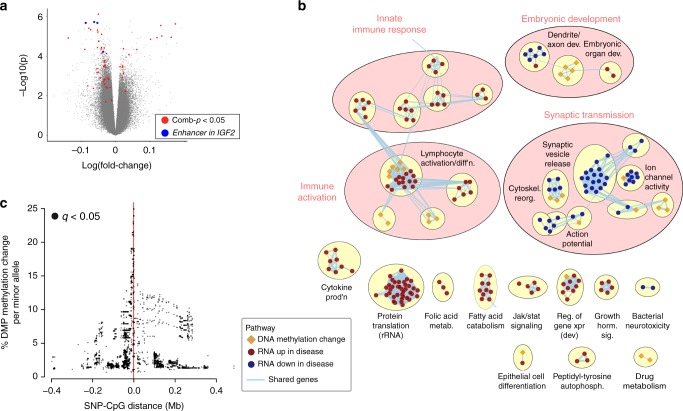
Epigenetic and transcriptomic alterations in frontal cortex neurons in major psychosis. **a** Volcano plot showing DNA methylation differences in neurons of major psychosis patients (*n* = 55 individuals) relative to controls (*n* = 27 individuals). Disease-specific DNA methylation changes were identified, after controlling for sex, age, post-mortem interval, and the first two genetic principal components (*n* = 406,332 probes). Red circles: Probes in regions with comb-p Šidák *p* < 0.05. Blue circles: Probes within the hypomethylated enhancer in *IGF2*. **b** Pathways significantly affected by DNA methylation and/or transcriptomic changes in major psychosis. Nodes show pathways enriched with differentially methylated regions in psychosis (diamonds; *q* < 0.05; hypergeometric test; *n* = 48 pathways of 6858 tested) or enriched in differentially expressed genes (circles; *q* < 0.05; GSEA preranked; *n* = 5580 pathways tested), and edges indicate common genes. Node fills indicate up- (red nodes; *n* = 183 pathways) or down- (blue nodes; *n* = 64) regulation in disease; epigenetic pathways indicate change in disease (orange diamonds). Clusters of similar pathways are grouped in pink circles (Enrichment Map, AutoAnnotate). Clusters with fewer than three nodes are not shown, unless these have both epigenetic and transcriptomic pathways. **c** Significant genetic-epigenetic *cis* interactions of differentially-methylated regions. The y-axis shows percent change in DNA methylation with increasing number of minor alleles, (*q* < 0.05; linear regression; *n* = 4762 interactions, 2212 SNPs with CpGs located in 18 differentially methylated regions)

We fine-mapped DNA methylation in neuronal nuclei (NeuN+) isolated by flow cytometry from post-mortem frontal cortex of the brain of individuals diagnosed with schizophrenia, bipolar disorder, and controls (*n* = 29, 26, and 27 individuals, respectively; Supplementary Data 1, Supplementary Table 1, Supplementary Fig. 1). We performed an epigenome-wide association analysis (EWAS) using Illumina MethylationEPIC microarrays surveying 812,663 CpG sites (Fig. 1 and Supplementary Figs. 2–4). In this analysis we controlled for age, sex, post-mortem interval, as well as genetic ancestry, which was determined by genotyping the same individuals (Infinium PsychArray-24 microarrays and imputed genotypes; 228,369 SNPs; *n* = 82 individuals; Supplementary Fig. 5). We identified 18 regions with significant DNA methylation changes in patients with major psychosis (comb-p Šidák *p* < 0.05; Fig. 1a; Supplementary Data 2; Supplementary Fig. 6a). Differentially methylated regions were enriched in pathways related to embryonic development, synaptic function, and immune cell activation (*q* < 0.05; hypergeometric test; Fig. 1b, Supplementary Data 3). We then determined the consequences of altered DNA methylation in major psychosis by profiling transcriptomes in a randomly selected subset of the same samples, by RNA sequencing (*n* = 17 cases, 17 controls; Supplementary Data 4, Supplementary Data 5, Supplementary Data 6, and Supplementary Figs. 6b and 7a), after adjusting for age, sex, post-mortem interval, and neuronal proportion. Pathway analysis revealed consistent alterations with those identified in the DNA methylation analysis, affecting early development, the innate immune system, and synaptic transmission (Fig. 1b). We further examined the developmental regulation of genes transcriptionally altered in psychosis, using the BrainSpan dataset. Pre- and post-natal transcriptional dynamics of genes differentially expressed in psychosis showed a significantly higher correlation with those of synaptic development genes, relative to randomly-sampled sets (BrainSpan; *p* < 0.001; resampling, one-sided test; Supplementary Fig. 7b). Together, these findings suggests that in neurons of major psychosis patients, DNA methylation and transcriptional changes converge to affect early development, disrupt neurotransmission, and raise immune responses.

### Genetic-epigenetic interactions in major psychosis neurons

We then identified genetic-epigenetic interactions at the differentially methylated regions in neurons of patients with psychosis. For this, we examined genotype information from the same individuals (82 individuals; Infinium PsychArray-24 microarrays) and imputed genotypes using 1000 Genomes reference panel, resulting in 228,369 SNPs (Supplementary Figs. 2 and 5). For each of the differentially-methylated regions, we performed a cis-meQTL analysis (which involves univariate SNP-CpG regression to assess the effect of genotype on base-level DNA methylation). We found that 13 of the 18 differentially methylated regions demonstrated significant genetic-epigenetic interactions in *cis* (*q* < 0.05; linear regression; 36 of 56 CpG probes within the 18 regions; 2212 of 13,552 SNPs in cis with differentially methylated probes) (Fig. 1c, and Supplementary Data 7). Additionally, one differentially methylated region at the *HLA* locus demonstrated significant genetic-epigenetic interactions with known genetic risk factors for schizophrenia^24,25^ (*q* < 0.05; linear regression; 4373 risk SNPs tested; Supplementary Data 8). Therefore, neurons of major psychosis patients show significant changes in DNA methylation, some of which may be mediated by genetic state.

### Hypomethylation of enhancer at *IGF2* in psychosis neurons

**Fig. 2:**
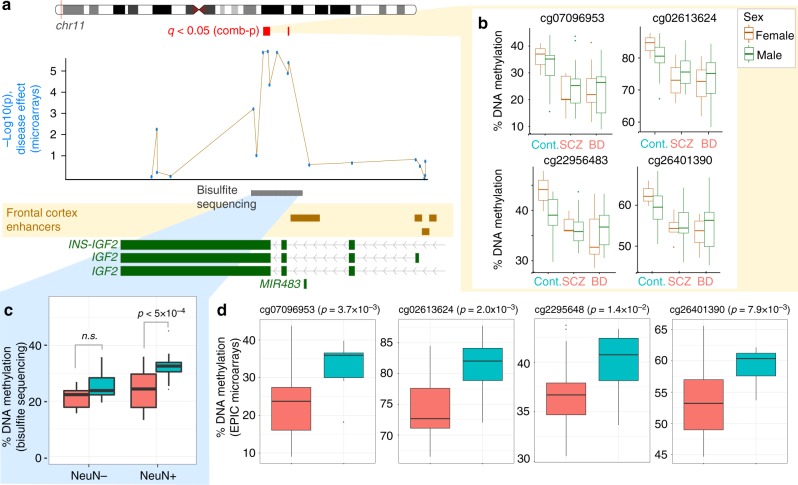
An enhancer at *IGF2* is differentially methylated in neurons of major psychosis patients. **a** Hypomethylation in psychosis at the *IGF2* locus. The view shows differentially methylated regions (red, comb-p) and nominal probe-level *p*-values (blue) in major psychosis cases (*n* = 55 individuals) relative to controls (*n* = 27 individuals), as identified by EPIC arrays. Also shown are adult frontal cortex enhancers (brown rectangles; NIH Roadmap Epigenomics Project) and region validated by targeted bisulfite sequencing (gray rectangle). **b** CpG probe-level methylation within differentially methylated *IGF2* region, by diagnostic subgroup and sex. Boxplot center indicates median; box bounds indicate 25th and 75th percentile, and whiskers mark 1.5 times the interquartile range. **c** Validation of *IGF2* hypomethylation using targeted bisulfite sequencing. Average % DNA methylation in a region in *IGF2* that is differentially methylated in major psychosis. Box plots show the % DNA methylation averaged over the ~1.3 kb enhancer region in neuronal DNA (NeuN+; 13 cases, 13 controls) and glial DNA (NeuN-: 10 cases, 12 controls). Bases with ≥10× coverage are included in the analysis. *P*-value from ANOVA for effect of disease, after controlling for age, sex, post-mortem interval, and batch effect. Base-level methylation estimates from EPIC arrays and targeted bisulfite sequencing were strongly correlated (Pearson’s coefficient *R* = 0.67, *p* < 10^−19^; *t*-test; Supplementary Fig. 9). Boxplot elements same as in **b**. **d** Validation of neuronal *IGF2* hypomethylation in cases, when samples are limited to males of European genetic ancestry (*n* = 25 cases, 11 controls). Nominal *p*-values from a nested ANOVA model for effect of diagnosis after controlling for age, post-mortem interval, and the first two genetic principal components. **b**–**d** Boxplot center indicates median; box bounds indicate 25th and 75th percentile, and whiskers mark 1.5 times the interquartile range. Boxplot elements same as in **b**

Notably, two of the top differentially methylated regions in major psychosis neurons were located at the 3′ end of the *IGF2* gene (Šidák *p* < 10^−3^; Fig. 2a; Supplementary Data 2a). Both schizophrenia and bipolar patients were consistently hypomethylated at the *IGF2* locus, relative to controls (3–9% probe-level hypomethylation in cases relative to controls in *IGF2* region; Fig. 2b). Hypomethylation of the *IGF2* locus was also observed in an analysis limited to individuals with genetic European ancestry (13 controls, 20 bipolar disorder, 19 schizophrenia; Šidák *p* < 2 × 10^−4^ for *IGF2* locus; Supplementary Data 2b). To assess the impact of lifestyle-related variables, we repeated probe-level tests for individual differentially methylated sites at the *IGF2* locus after controlling for smoking status (ever/never) and reported antipsychotic use (some/none), in addition to age, sex, post-mortem interval, and the first two genetic principal components. The *IGF2* locus remained significantly hypomethylated in neurons of patients with major psychosis even after accounting for these lifestyle-related covariates (*p* < 0.05; nested ANOVA; DNA methylation for effect of disease relative to individual covariates in Supplementary Fig. 8). Furthermore, we did not find evidence of *cis*-acting genetic-epigenetic effects for any of the probes in the differentially methylated *IGF2* region (*q* > 0.05; Supplementary Data 9).

We also confirmed the reliability of the Illumina MethylationEPIC array findings by fine-mapping DNA methylation at the *IGF2* genomic area (~161 kb) in neurons, using a targeted bisulfite sequencing assay (*n* = 13 cases, 13 controls; array and bisulfite sequencing methylation correlation *R* = 0.67, *p* < 10^−19^; Supplementary Fig. 9). This analysis also defined the *IGF2* site as being a 1.3 kb region with significant hypomethylation in neurons of major psychosis cases (7.4% hypomethylation *p* < 5 × 10^−4^; nested ANOVA model; effect of disease after controlling for age, sex, post-mortem interval, and batch effect; Fig. 2c, Supplementary Data 10). In addition, we performed targeted bisulfite sequencing of the *IGF2* enhancer locus in glial cells (NeuN-) isolated from the same individuals (*n* = 10 cases, 12 controls). While we observed a similar trend of disease-specific hypomethylation in glial cells, this effect was not significant (4% hypomethylation; *p* = 0.07; nested ANOVA model; Fig. 2c).

To further verify that our effect is not confounded by sex and ethnicity, we reanalyzed our dataset examining only males of European genetic ancestry. Significant *IGF2* hypomethylation persisted in three of four tested CpG probes when samples were limited to males of European genetic ancestry (*n* = 25 cases, 11 controls; Bonferroni-corrected *p* < 0.01; nested ANOVA model; effect of disease after accounting for age, post-mortem interval, and first two principal components of genetic ancestry; Fig. 2d).

### Dopamine synthesis abnormalities linked to enhancer at *IGF2*

**Fig. 3:**
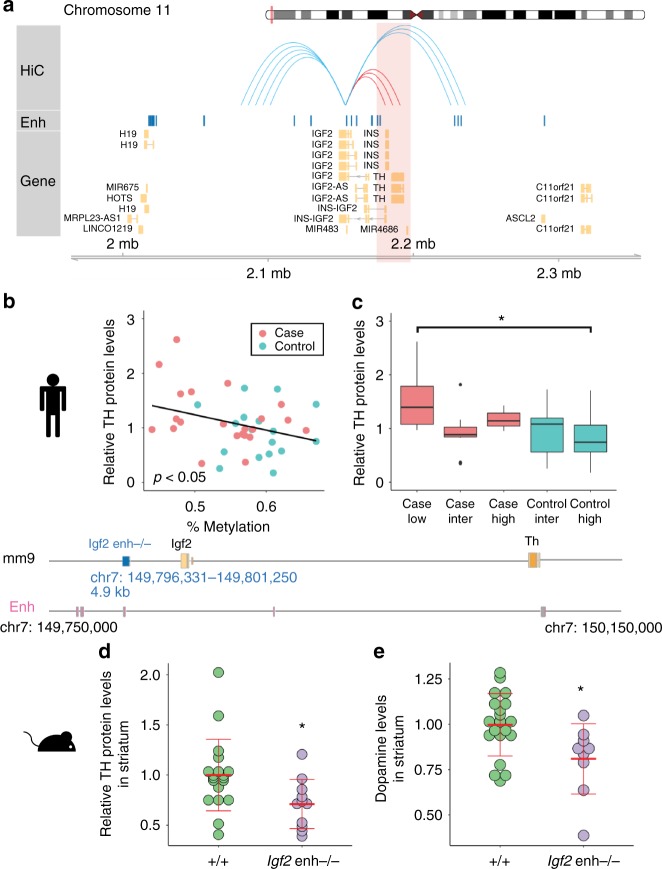
The differentially methylated enhancer at *IGF2* in major psychosis targets the dopamine synthesis enzyme tyrosine hydroxylase (*TH*). **a** Higher-order chromatin interactions of the differentially-methylated enhancer at *IGF2* with the *TH* gene in the human prefrontal cortex^90^; interactions within ±100 kb are shown. Blue arcs show all interactions from the location, and red arcs highlight those to the *TH* gene. Enh: Chromatin states reflecting enhancers in adult frontal lobe^91^ (blue rectangles). **b** Reduced DNA methylation at the *IGF2* enhancer in major psychosis correlates with increased TH protein levels in the prefrontal cortex (*R* = −0.32, *p* < 0.05; linear regression; *n* = 17 controls (blue circles) and *n* = 22 cases (red circles)). TH protein levels are normalized to NeuN, INA, and actin. **c** DNA methylation at the *IGF2* enhancer is associated with differing TH protein levels between cases and controls (same data as **b**). Low, mid, or high DNA methylation ( < 50%, 50–60%, and > 60%, respectively). Left to right: *n* = 8, 11, 3, 9, and 8 samples for cases (red boxplots) and controls (blue boxplots). Main effect of DNA methylation by two-way ANOVA *F*_(2, 35)_ = 3.5, *p* < 0.05; *p = 0.05 by Tukey post-hoc test. Boxplot center indicates median; box bounds indicate 25th and 75th percentile, and whiskers mark 1.5 times the interquartile range. **d**, **e** Effect of *Igf2* enhancer deletion knockout in mice. Schema shows deletion of the 4.9 kb *Igf2* enhancer alongside mouse forebrain enhancers (ENCODE^92^, pink). **d** TH protein levels in striatum of adult wild-type (+/+; *n* = 19 mice; green circles) and *Igf2*enh−/− (*n* = 11 mice; purple circles) mice (normalized to NeuN and actin). Data points shown along with mean±standard deviation. **e** Dopamine levels in striatum of adult wild-type and *Igf2*enh−/− mice measured by HPLC (*n* = 20 and 9 mice, respectively). Data normalized to wild-type levels. *p < 0.05 by one-way ANOVA

The hypomethylated *IGF2* locus in major psychosis overlapped an enhancer in the adult frontal cortex (Fig. 2a; data from NIH Roadmap Epigenomics Project). Assessment of chromatin interactions in the prefrontal cortex by analysis of Hi-C data revealed that this enhancer targets the tyrosine hydroxylase (*TH*) gene promoter (Fig. 3a; Supplementary Fig. 10). TH is the rate-limiting enzyme for the production of the neurotransmitter dopamine. Dopamine dysregulation in the cortex and striatum of both patients with schizophrenia and bipolar disorder is centrally involved in the cognitive and psychotic symptoms of these diseases^26,27^. Reduced DNA methylation at the enhancer in *IGF2* was associated with elevated levels of TH protein levels in the human frontal cortex (*R* = −0.32, *p* < 0.05; linear regression; Fig. 3b, c), supporting the hypothesis that this enhancer modulates dopamine synthesis. Accordingly, the top differentially expressed genes from the transcriptomic profiling described above – namely, *NR4A1*, *NR4A2*, and *EGR1* – are transcription factors that affect *TH* and *IGF2* expression^28–33^ (STRING database interactions, Supplementary Fig. 7c), supporting dysregulation of the *TH*-*IGF2* locus in major psychosis.

### *Igf2* enhancer loss affects dopamine levels and synapses

We then examined transgenic mice carrying an intergenic *Igf2* enhancer deletion (Fig. 3). Since the intergenic enhancer region we deleted in mice is near the *Igf2* gene but may not be the ortholog of the human *IGF2* enhancer, we first analyzed Hi-C data of mouse cortical neurons, which showed that this mouse enhancer does target the promoter of the *TH* gene as well as the *Igf2* gene (Supplementary Fig. 11). In these mice, we examined the frontal cortex and striatum, the latter being a major site of dopamine production in the brain. In the striatum, inactivation of the *Igf2* enhancer led to a decrease in TH protein levels and in dopamine (*p* < 0.05; one-way ANOVA; Fig. 3d, e); this effect was not observed in the frontal cortex (Supplementary Fig. 12). TH protein levels are 5.6-fold greater in the mouse striatum relative to frontal cortex (*p* < 10^−11^; one-way ANOVA; Supplementary Fig. 13), which may explain the capacity to detect a decrease in striatal, but not frontal, TH in mice lacking the enhancer at *Igf2*. These data collectively suggest that in schizophrenia and bipolar disorder, epigenetic disruption of enhancer activity at the *IGF2* locus in neurons leads to abnormalities in subcortical dopaminergic signaling, which is centrally involved in the development of psychotic symptoms.

**Fig. 4:**
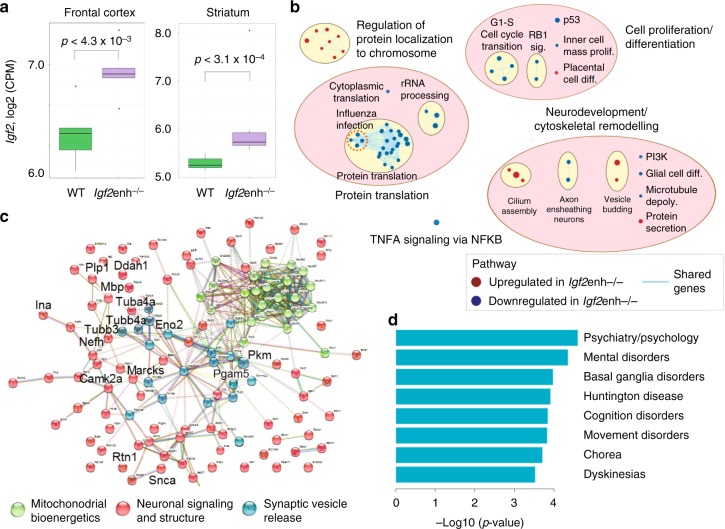
Transcriptomic and synaptic proteome alterations in the brain of mice lacking the enhancer at *Igf2.* **a** Transcript levels of *Igf2* in the frontal cortex and striatum of adult wild-type and *Igf2*enh−/− mice. Left: frontal cortex (green boxplots: *n* = 6 wild-type, purple boxplots: 6 *Igf2*enh−/− mice). Right: striatum (*n* = 7 wild-type mice, 8 *Igf2*enh−/− mice). *P*-values from two-sided Wilcoxon test. Boxplot center indicates median; box bounds indicate 25th and 75th percentile, and whiskers mark 1.5 times the interquartile range. **b** Pathways enriched for differential expression in the striatum of *Igf2*enh-/- mice, relative to wild-type mice. Nodes shows pathways with *q* < 0.05 (68 pathways, preranked GSEA) and edges indicate shared genes (EnrichmentMap^87^; default setting of Jaccard and overlap = 0.375). Red nodes: upregulated expression in *Igf2*enh−/−; blue nodes: downregulated expression in *Igf2*enh−/− mice. Pathways were clustered using AutoAnnotate^93^. Full pathway enrichment results in Supplementary Data 15. **c** Functional annotation of synaptic proteins significantly altered in the striatum of *Igf2*enh−/− mice relative to wild-type mice (*q* < 0.01; one-way ANOVA; *n* = 6 wild-type mice, 6 *Igf2*enh−/− mice, 2 pools per genotype with 3 mice/pool). Genes overlapping synaptosomal proteome abnormalities in schizophrenia brain^36^ are highlighted. Quantitative proteome analysis by mass spectrometry and clustering of top proteins altered in *Igf2*enh−/− mice by STRING. Node fills indicate gene pathways; green: mitochondrial bioenergetics; red: neuronal signaling and structure; blue: synaptic vesicle release. **d** Disease pathways enrichment for synaptic proteins dysregulated in *Igf2*enh−/− mice (*q* < 0.05, MetaCore; 715 pathways tested; Supplementary Data 17)

We further examined the widespread consequences of enhancer disruption at the *Igf2* locus in the brain by profiling the transcriptome. We used RNA-sequencing to assess the transcriptomes of wild-type and *Igf2* enhancer deletion mice, examining the frontal cortex and striatum (Supplementary Data 11; Supplementary Fig. 14). Enhancer deletion resulted in a significant upregulation of *Igf2* expression in both the frontal cortex and striatum of *Igf2*enh−/− mice (Fig. 4a; *p* < 4.3 × 10^−3^ in frontal cortex, *n* = 6 wild-type, 6 *Igf2*enh−/− mice; *p* < 3.1 × 10^−4^ in striatum, *n* = 7 wild-type, 8 *Igf2*enh−/− mice; two-sided Wilcoxon–Mann–Whitney test). In total, there were 232 and 56 genes that were differentially expressed (*q* < 0.05; generalized linear regression by edgeR^34^) in the frontal cortex and striatum, respectively (effect of genotype, after controlling for sex; Supplementary Data 12 and 13). Pathway enrichment analysis identified that *Igf2* enhancer deletion resulted in alterations in cell proliferation/development, protein synthesis, immune responses, neurodevelopment, and cytoskeletal remodeling (*q* < 0.05; GSEA;^35^ 143 and 68 of 6321 pathways tested for frontal cortex and striatum, respectively; Fig. 4b; Supplementary Data 14 and 15; Supplementary Fig. 15). Notably, the pathway reflecting TNF-alpha signaling via NF-kB was a top-ranking pathway (*q* < 0.005; GSEA;^35^ Supplementary Data 14 and 15), which is consistent with prior reports that *Igf2* modulates synaptic plasticity via NF-kB signaling^16^. To further explore synaptic alterations induced by *Igf2* enhancer deletion, we performed a proteomic analysis of synaptosomes from the striatum of wild-type and *Igf2*enh−/− mice using quantitative mass spectrometry (Supplementary Figs. 16 and 17). We discovered widespread changes in *Igf2*enh−/− mice relative to wild-type mice; 956 of 3619 proteins tested were significantly different (*q* < 0.05; one-way ANOVA; Supplementary Data 16). Synaptic proteins with the highest change were involved in neurosignaling and structure, mitochondrial bioenergetics, and synaptic vesicle release (*q* < 0.01; hypergeometric test; Fig. 4c). Several proteins altered by *Igf2* enhancer deletion had been found dysregulated in the synaptosomal proteome of schizophrenia patients^36^, including genes affecting synaptic plasticity and neurotransmitter release, such as calcium/calmodulin dependent protein kinase II alpha (*Camk2a*), myristoylated alanine-rich C-kinase substrate (*Marcks*), and alpha-synuclein (*Snca*) (Fig. 4c). The top disease pathways enriched in striatal synaptosomes of mice lacking the enhancer at *Igf2* were related to psychiatric, mental, and movement disorders (*q* < 0.05; hypergeometric test; 8 pathways of 715 tested for genes with *q* < 0.01; Fig. 4d; Supplementary Data 17 and 18). Therefore, loss of the enhancer at *Igf2* in mice disrupts synaptic proteins involved in neurotransmission and associated with psychiatric disease.

## Discussion

In sum, we identified a decrease in repressive epigenetic marks at an enhancer linked to *TH* gene regulation in neurons of patients with major psychosis. Enhancer-mediated upregulation of TH, promoting higher striatal dopamine synthesis, would augment the risk for psychosis^26^. Hence, hypomethylation of the enhancer at *IGF2* may be an important contributor to the pathogenesis of psychotic symptoms.

Interestingly, in patients, the progressive loss of prefrontal cortex volume closely parallels the development of psychosis^21,22^. Imaging studies of at-risk individuals show greater prefrontal cortical volume loss in individuals that transition to psychosis compared to those remaining healthy^22^. The severity of psychotic symptoms is also associated with structural alterations in the cortex^23^. This link between psychotic symptoms and brain development may involve the molecular regulation of the *IGF2* locus identified in this study. In the brain, *IGF2* promotes synapse development, spine maturation, and memory formation^16–19^, signifying that normal *IGF2* activation is required for healthy neuronal architecture. Recently, *IGF2* was found to be the top downregulated gene in the schizophrenia prefrontal cortex in the large CommonMind consortium RNA-sequencing study^37^. Loss of DNA methylation at the *IGF2* locus has been associated with decreased *IGF2* mRNA levels in early development^38^, and risk factors for schizophrenia; prenatal exposure to famine^15^ and reduced brain weight^39^. Similarly, our transcriptome analysis in major psychosis patients found a downregulation of genes affecting synaptic transmission and interacting with *IGF2*. In support, mice lacking the enhancer at *Igf2* had a decrease in TH and dopamine levels, along with an *Igf2* upregulation as well as transcriptomic and proteomic alterations affecting synaptic activity and structure. Therefore, in neurons of major psychosis patients, epigenetic changes facilitating a recruitment of the enhancer at *IGF2* for activation of *TH*, may, in tandem, impede *IGF2* regulation. We propose that improper epigenetic control of an *IGF2* enhancer may simultaneously contribute to dopamine-mediated psychotic symptoms and synaptic structural deficits in major psychosis.

A limitation to this study is that inter-species differences in enhancer size and location makes it challenging to demonstrate equivalence of human and mouse enhancers^40^. Nonetheless, our findings demonstrate that altered activity of the enhancer nearest to the *Igf2* gene in the mouse affects TH protein levels and changes gene expression in pathways affecting neurodevelopment, neurosignaling, and synaptic activity, as was observed in major psychosis patients with a hypomethylated enhancer at *IGF2*. This shared consequence in humans and in transgenic mice supports the hypothesis that *IGF2* enhancer activity is associated with altered TH regulation and dopamine synthesis. Further study is required to fully characterize the extent to which enhancers regulate *TH* and dopamine signaling in psychotic disorders.

The multi-omics approach in isolated neurons used in this study offers a rich dataset for investigating the molecular events involved in major psychosis. Many of the epigenetic abnormalities identified in major psychosis neurons were associated with genotype, suggestive of a genetic origin and the potential that these epigenetic states may be set early in life, before the onset of disease symptoms. However, our findings do not preclude the role of, and interaction with, non-shared environmental factors, particularly during early synaptic development^4^ and in response to environmental stressors like inflammation^41^. It will be also important to replicate these findings in a second large cohort of major psychosis and control neurons. Future genome-scale studies, expanding this dataset to other types of epigenetic modifications (i.e., non-CG methylation, hydroxymethylation, histone marks) and to neurons of other brain regions will also be important for understanding the dynamic interplay between epigenome, transcriptome, and genetic factors in major psychosis. Of particular interest will be studies examining whether hypomethylation at the *IGF2* enhancer extends from a risk factor to a prognostic marker in peripheral tissues for the development of psychosis.

## Methods

### Human tissue samples

Post-mortem brain samples of frontal cortex were obtained through the NIH NeuroBioBank at the University of Pittsburgh; the Harvard Brain Tissue Resource Center; the Human Brain and Spinal Fluid Resource Center at Sepulveda; and the University of Miami Brain Endowment Bank. Patient data is provided in Supplementary Data 1. We obtained sample information on demographic factors (age, sex), clinical variables (cause of death, medications at time of death, duration of antipsychotic use, smoking status, and brain weight), and tissue quality (post-mortem interval, tissue quality/RIN score). Our analyses controlled for sample age, sex, post-mortem interval, ethnicity, and the influence of the clinical and technical covariates was examined in our data. The study protocol was approved by the institutional review board at the Centre for Addiction and Mental Health and the Van Andel Research Institute (IRB #15025).

### Lifestyle-related factors

Lifestyle factors were coded in the same manner for cases and controls. To ascertain which patients had a history of antipsychotic treatment, we used the following approach: FDA-approved antipsychotics were collected from the literature (https://www.fda.gov/Drugs/DrugSafety/ucm243903.htm^42^), including generic and brand names. Patient medication information was computationally searched for keyword matches from this list to identify drugs used by individuals; the mood stabilizers lithium and valproic acid were included in this list. Where no match was found, antipsychotic status was set to none. Where we controlled for antipsychotic treatment, patients were divided into those who ever had antipsychotic use and those who did not. Smoking status was similarly binarized, so that any lifetime record of smoking resulted in a categorization of the sample as a smoker or non-smoker (i.e. ever or never). Individuals with missing information were not included in the analysis examining the effects of lifestyle factors.

### Isolation of neuronal nuclei using flow cytometry

Neuronal nuclei were separated using a flow cytometry-based approach, similar to as described^43,44^. Briefly, human brain tissue (250 mg) for each sample was minced in 2 mL PBSTA (0.3 M sucrose, 1X phosphate buffered saline (PBS), 0.1% Triton X-100). Samples were then homogenized in PreCellys CKMix tubes with a Minilys (Bertin Instruments) set at 3,000 rpm for three 5 s intervals, 5 min on ice between intervals. Samples homogenates were filtered through Miracloth (EMD Millipore), followed by a rinse with an additional 2 mL of PBSTA. Samples were then placed on a sucrose cushion (1.4 M sucrose) and nuclei were pelleted by centrifugation at 4000 × g for 30 min 4 °C using a swinging bucket rotor. For each sample, the supernatant was removed and the pellet was incubated in 700 μl of 1X PBS on ice for 20 min. The nuclei were then gently resuspended and blocking mix (100 μl of 1X PBS with 0.5% BSA (Thermo Fisher Scientific) and 10% normal goat serum (Gibco) was added to each sample. NeuN-488 (1:500; Abcam; ab190195) was added and samples were incubated 45 min at 4°C with gentle mixing. Immediately prior to flow cytometry sorting, nuclei were stained with 7-AAD (Thermo Fisher Scientific) and passed through a 30 μM filter (SystemX). Nuclei positive for 7-AAD and either NeuN + (neuronal) or NeuN- (non-neuronal) were sorted using an Influx (BD Biosciences) or BD FACSAria IIIu (BD Biosciences) at the Faculty of Medicine Flow Cytometry Facility (Toronto, ON, Canada). Approximately 1 million NeuN+nuclei were sorted for each sample. Immediately, after sorting nuclei were placed on ice and then precipitated by raising the volume to 10 mL with 1X PBS and adding 2 mL 1.8 M sucrose, 50 μl 1 M CaCl_2_ and 30 μl Mg(Ace)_2_ and centrifugation at 1786 × *g* for 15 min at 4 °C. The supernatant was removed from NeuN+ and NeuN– samples and pellets were stored at −80 °C. Genomic DNA from each NeuN+ and NeuN− fraction of each sample was isolated using standard phenol-chloroform extraction methods.

### Genome-wide DNA methylation profiling

Whole-genome DNA methylation profiling for each sample was performed on Illumina MethylationEPIC BeadChip microarrays at The Centre for Applied Genomics (Toronto, Canada). Bisulfite converted DNA samples (*n* = 104) were randomized across arrays (8 samples/array). Data generated from the microarrays were preprocessed with Minfi v1.19.12 (software details listed in Supplementary Note 1). Normalization was performed with noob^45^, followed by quantile normalization. We confirmed that the sex of the individuals, as identified from the genotype data (described below) matched that inferred from the DNA methylome (minfi getSex() function). Probes that overlapped SNPs (minor allele frequency >0.05) on the CpG or single-base extension were excluded (11,812 probes), as were probes known to be cross-reactive^46^ (42,558 probes) and those that failed detectability (*p* > 0.01) in > 20% samples (1170 probes). After processing, 812,663 probes were left. Principal component analysis (PCA) was performed on the matrix of beta values and the first three principal component projections were examined for all samples; samples were color-coded in turn by various biological and technical variables (Supplementary Fig. 3). Based on this PCA co-clustering, one sample, despite being labeled NeuN+ was an outlier; this sample was excluded from downstream analyses. PCA plots revealed no sample separation by the array slide on which samples were run. We provided the surrogate variable analysis^47^ calculator with the known covariates of age, sex, diagnosis, and post-mortem interval, and the model identified no additional surrogate variables. Additionally, we did not observe structure in the data exploration (PCA, hierarchical clustering, Supplementary Figs. 3 and 4), suggesting there is no major unknown confounder. Therefore, we conclude that there are no major sources of unexplained variation.

We used the BioConductor package bacon^48^ to compute lambda for our EWAS, providing it with t-statistics from the main EWAS reported in our manuscript (inflation() function; 82 samples; age, sex, post-mortem interval, and first two genetic principal components were included as covariates). The estimated inflation factor is 1.03, which is the regime of minimal inflation for an EWAS (<1.14^48^).

### Analysis of differentially methylated regions

The top 50% probes with highest variance were used to identify differentially methylated probes (406,332 probes). For each probe, a linear model was fit using the R package *limma*, with technical replicates treated as blocking factors and by applying variance shrinkage with an empirical Bayes approach^49^; in addition, diagnosis, age, sex, post-mortem interval, and the first two principal components of genetic ancestry were used as covariates. Benjamini–Hochberg FDR correction was used to correct nominal *p*-values. Principal components of genetic ancestry were computed using plink^50,51^, using genotypes from the same patients (see Genotype data processing section below). Sample identity was confirmed by comparing inferred genotypes from EPIC array SNP probes, to those of overlapping SNPs in the genotyping arrays (Supplementary Fig. 5). Genotypes were inferred from EPIC SNP probes by fitting a 3-component mixture model to SNP beta values (https://github.com/ttriche/infiniumSnps). Adding to the linear model described above, we also performed a sensitivity analysis that included microarray slide as a covariate, and found that this variable did not alter the results. As principal component plots also showed no sample separation based on array slide, this term was not included in the final model. Probe-level *p*-values were grouped into clusters of differentially methylated regions using the Python module Comb-p^52^; comb-p groups spatially correlated differentially methylated probes (seed *p*-value of 0.01 to start a region, at a maximum distance of 500 bp in each brain tissue, as reported^53^). The *p*-values for differentially methylated regions were corrected for multiple testing using Šidák correction. The Šidák method of multiple correction is proposed as part of the comb-p algorithm^52,54^. It is a powerful alternative to Bonferroni correction, as it uses a corrected alpha value of alpha_0_ = 1-(1-alpha)^1/k^ (where “alpha” is the alpha for each test). This increases the alpha_0_ as the number of tests (k) increases. All analyses were performed in R v3.3.1 or 3.4.0.

### Targeted bisulfite sequencing at *IGF2* locus

DNA methylation at the *IGF2* and surrounding genomic area (161 kb) was captured using the SeqCap Epi Enrichment System (Roche). Biotinylated long oligonucleotide probes targeting 450 sites at the extended *IGF2* locus (unique, non-repetitive genome) were custom designed by Roche NimbleGen. Library preparation, done in two batches, was performed following manufacturer instructions. Briefly, gDNA (500 ng) of each sample (*n* = 15 controls and 14 cases, with four technical replicate samples for NeuN+; 12 controls and 10 cases for NeuN-) were fragmented (~200 bp), end repaired, and ligated to barcoded adapters using the KAPA Library Preparation kit (Kapa Biosystems) and SeqCap Adapter Kit A and B (Roche). Bisulfite conversion of the adapter ligated DNA, followed by column purification, was performed with the EZ DNA Methylation Lightning kit (Zymo). The bisulfite converted DNA for each sample was then amplified by ligation mediated PCR (95 °C for 2 min, 10 cycles of [98 °C for 30 s, 60 °C for 30 s, 72 °C for 4 min], 72 °C 10 min, 4 °C hold) followed by purification with Agencourt AMPure XP beads (Beckman Coulter). Sample quality was verified on a Bioanalyzer (Agilent) and quantity was determined with a NanoDrop spectrophotometer (Thermo Fisher Scientific). Equimolar amounts of each sample were then combined into a single pool. The *IGF2* target region was captured by hybridizing the amplified bisulfite converted DNA pool (1 μg) to the probe library (Roche), as directed by manufacturer. Enrichment and recovery of captured bisulfite-converted DNA was completed by binding to magnetic beads and subsequent wash steps using the SeqCap Pure Capture Bead kit and the SeqCap Hybridization and Wash kit (Roche). The captured DNA was then amplified by ligation mediated PCR (98 °C for 45 s, 11 cycles of [98 °C for 15 s, 60 °C for 30 s, 72 °C for 30 s], 72 °C for 1 min) followed by purification with Agencourt AMPure XP beads (Beckman Coulter). Library quality and quantity was assessed using a combination of Agilent DNA High Sensitivity chip on a Bioanalyzer (Agilent Technologies), Qubit dsDNA HS Assay kit on a Qubit 3.0 fluorometer (Thermo Fisher Scientific), and Kapa Illumina Library Quantification qPCR assays (Kapa Biosystems). DNA sequencing was performed on an Illumina HiSeq 2500 on Rapid Run mode, and on an Illumina NextSeq 500 sequencer.

Data were processed using the pipeline recommended by the manufacturer^55^. Trimmomatic^56^ was used to trim read adapter sequences and BSMAP^57^ was used to align reads to the GRCh38/hg38 genome build. The genome index consisted of reference chromosome sequences (chromosomes 1-22, X, Y, and M) and the lambda phage genome (https://www.ncbi.nlm.nih.gov/nuccore/215104). Following alignment, reads were pooled for each sample. The merged set of reads was separated into those aligning to top and bottom strand, duplicates were removed with Picard, and then matching read pairs were merged. Samtools^58^ was used to exclude reads that were not properly paired or were unmapped. Bamutils were used to clip overhanging reads that distort methylation estimates. Methratio.py in BSMAP computed the percent methylation at the base level. Two samples contained spiked-in lambda phage DNA; these showed that bisulfite conversion efficiency exceeded 99%.

Only bases with at least 10x coverage were included in locus-level analyses. Using all bases overlapping a given locus, locus-level methylation was calculated as the sum of all C counts divided by the sum of all (C+T) counts. Locus-level methylation was averaged across technical replicates. Locus-level differences were ascertained using a nested ANOVA model. Statistical significance was ascertained with an F-test comparing a full model that includes diagnosis, over the null model explaining effect by age, sex, and post-mortem interval (PMI). Data exploration showed a batch effect, therefore a batch term was included in the null model.

$${\mathrm{Null}}\,{\mathrm{model:}}\,M = {\mathrm{age + sex + PMI + batch + error}}$$

$${\mathrm{Full}}\,{\mathrm{model:}}\,M = {\mathrm{DX + age + sex +PMl + batch + error}}$$

### Genotype data processing

SNPs in each sample (*n* = 99 samples, comprising of 83 biological replicates) were determined using the Infinium PsychArray-24 processed by The Centre for Applied Genomics (Toronto, Canada). Samples were randomized across SNP arrays. LiftOver was used to convert genotypes to the GRCh37/hg19 build. Quality control was performed as described^59^. SNPs with a minor allele frequency <0.05, those with HWE *p* < 10^−6^ and those missing in > 1% samples were excluded. Where pairs of individuals had relatedness (Identity By State; IBS) > 0.185, one was excluded. Samples with < 90% SNPs genotyped and those with outlier heterozygosity were excluded. 98 samples (82 biological replicates) and 228,369 SNPs passed quality control. Principal components of genotype data, which represent genetic ancestry and are used as covariates for the EWAS and meQTL analysis, were extracted using plink^50,51^ on study samples. Continental genetic ancestry was ascertained by multidimensional scaling using HapMap3 as a reference population^60^. For European-specific EWAS, Europeans were defined as individuals with MDS 1 and 2 lying within 3 standard deviations of the mean defined by the CEU population in the HapMap3 reference panel. The biological sex of samples was confirmed by matching the sex ascertained from the genotype data to that using control probes on the methylation arrays (minfi getSex() function).

As a measure of the extent of population stratification, we computed lambda using the genotype data (82 samples, technical replicates excluded) for a plink logistic regression on case/control status, after adjusting for age, sex, post-mortem interval, and the first two genetic principal components, and the lambda is 1.07; this value is in the regime of acceptable values for GWAS studies (~1.05^61^).

### meQTL analysis

96 samples (82 biological replicates) passed genotype processing quality control and were used for meQTL analysis. For meQTL analysis, we first imputed genotype data using Check-bim and the Michigan Imputation Server^62^ (Eagle v2.3;^63^ 1000G^64^ Phase 3 v5; Population:ALL). SNPs with a conservative imputation INFO score of >0.7 were retained (INFO score is a measure of confidence in imputed genotype call). For cis e-QTL inference, SNPs within ±500 kb of CpG probes in differentially-methylated regions were tested. For trans e-QTL inference, SNPs from schizophrenia GWAS study^24^ (SNPs with nominal *p* < 10^−9^), and SCZ credible SNPs were included^24,25^. Only SNPs with ≥10 individuals per genotype were tested (each tested SNP has ≥10 samples with AA, ≥10 samples with AB, and ≥10 samples with BB). This conservative threshold was set to identify high-confidence SNP-CpG interactions, and is comparable to those previously described^8,53,65^. A linear regression was used to assess the effect of genotype on DNA methylation, with sex, diagnosis, age, and the first two genetic principal components as covariates. DNA methylation for technical replicates was averaged, and where technical replicates existed for genotype, the first replicate was used. Benjamini–Hochberg correction was applied for multiple testing with statistical significance at *q* < 0.05.

### Gene expression profiling by RNA-seq

We performed RNA-seq on 17 cases and 17 controls (*n* = 34 human samples) from the total samples analyzed in the DNA methylation study. These randomly selected samples for RNA-seq had a similar DNA methylation status at the *IGF2* locus in cases relative to controls, as the full cohort (5–9% hypomethylation in cases). Prefrontal cortex samples were lysed using QIAzol Lysis Reagent (Qiagen) and homogenized with a TissueLyser (Qiagen). Total RNA from each sample was isolated using the RNeasy Plus Universal Mini kit (Qiagen) according to manufacturer’s instructions and included an enzymatic DNase (Qiagen) digestion step. RNA quality was measured on a 2100 Bioanalyzer (Agilent) and quantity was determined with a Nanodrop 2000 spectrophotometer (Thermo Fisher Scientific). RNA samples had a RIN quality score >7 and proceeded to RNA-seq library preparation (RIN between 7.1 and 9.4 for all samples). Libraries were prepared by the Van Andel Genomics Core from 300 ng of total RNA using the KAPA RNA HyperPrep Kit with RiboseErase (v1.16) (Kapa Biosystems). RNA was sheared to 300-400 bp. Prior to PCR amplification, cDNA fragments were ligated to Bio Scientific NEXTflex Adapters (Bioo Scientific). Quality and quantity of the finished libraries were assessed using a combination of Agilent DNA High Sensitivity chip (Agilent Technologies, Inc.), QuantiFluor® dsDNA System (Promega Corp.), and Kapa Illumina Library Quantification qPCR assays (Kapa Biosystems). Individually indexed libraries were pooled, and 75 bp paired-end sequencing was performed on an Illumina NextSeq 500 sequencer, with all libraries run across 3 flow cells. Base calling was done by Illumina NextSeq Control Software (NCS) v2.0 and output of NCS was demultiplexed and converted to FastQ format with Illumina Bcl2fastq v1.9.0.

Trimgalore (v0.11.5) was used for adapter removal prior to genome alignment. STAR^66^ (v2.3.5a) index was generated using Ensemble GRCh38 p10 primary assembly genome and the Gencode v26 primary assembly annotation. Read alignment was performed using a STAR two-pass mode. To match genotypes between RNAseq and PsychArray-24, GATK Haplotype caller was applied to extract SNPs from aligned bam files, following the best practices instruction from the GATK website (https://gatkforums.broadinstitute.org/gatk/discussion/3892/the-gatk-best-practices-for-variant-calling-on-rnaseq-in-full-detail). Plink^50,51^ was used to convert VCF files to bed/bim/fam files. LiftOver was used to convert PsychArray genotype array coordinates from hg19 to the hg38 build used in the RNA-seq data. SNPs in common with the unimputed genotypes were identified (~11.4K SNPs) and extracted using plink^50,51^. SNP call overlap was computed as the number of SNPs for which number of minor alleles (0, 1, or 2) was identical between the genotype and RNA-seq platforms. We found perfect sample matching between the RNA-seq and genotype platforms (*n* = 30 samples tested; median of ~5.2K SNPs tested; 93-96% genotype match; Supplementary Fig. 5b).

Gene counts matrix was imported into R (3.4.1) and low expressed genes (counts per million (CPM) <1 in all samples) were removed prior to differential expression in edgeR^34^. Gene counts were normalized using the trimmed mean of *M*-values, fitted in a generalized linear model and differentially tested using a likelihood ratio test. The generalized linear model included  age, sex, post-mortem interval, and neuronal cell composition as covariates. Cell-type compositions for each sample was accessed using CIBERSORT^67^ on normalized sample counts against cell-type specific markers (see below), identifying the proportion of neurons in each samples. Benjamini-Hochberg correction was used to adjust for multiple testing. We also performed a sensitivity analysis to confirm that genetic ancestry did not alter our RNA-seq findings, and found that analysis of only individuals with European ancestry (exclusion of 3 non-European individuals) had strongly correlated results (Pearson correlation = 0.91) and the same top gene hits as the original analysis.

Our RNA-seq analysis corrected for the proportion of neuronal cells in each sample. Neuronal cell proportions were determined by CIBERSORT^67^ (http://cibersort.stanford.edu), which involved a gene signature matrix derived from single cell RNA-seq measures in adult human brain cells (signature matrix;^68^ source^69^). Because major psychosis is characterized by a loss of synaptic density, we excluded genes encoding synaptic proteins (Genes2Cognition database;^70^ lists L00000009, L00000016, and L00000012) from the gene signatures. One hundred and thirty-five synapse-associated genes were excluded, leaving 768 genes in the deconvolution analysis. CIBERSORT was run (100 permutations), and the inferred proportion of neurons was used as a covariate for differential expression.

### Pathway enrichment analysis

Pathways affected by the DNA methylation and transcriptomic changes in major psychosis were determined. For DNA methylation data, probes were mapped to genes if they overlapped between 1 kb upstream of the transcription start site to the transcription end site. Gencode^71^ v27 (liftOver to GRCh37) were used for gene extents. Pathway definitions were aggregated from HumanCyc^72^, IOB’s NetPath^73^, Reactome^74,75^, NCI Curated Pathways^76^, mSigDB^35^, Panther^77^, and Gene Ontology^78,79^. The same pathway sets were used for the DNA methylation and transcriptomic analysis. For DNA methylation pathway analysis, only pathways with 10–500 genes were included (6858 pathways). For pathway analysis of DNA methylation, a hypergeometric test was performed comparing the proportion of foreground probes (*p* < 0.05 from DNA methylation region analysis) to background probes (all probes tested in DNA methylation region analysis). Pre-ranked GSEA^35^ was used for transcriptomic pathway analysis, as it separates pathways upregulated in disease from those downregulated in disease (see Gene expression profiling by RNA-seq section for details). Benjamini-Hochberg correction was performed to adjust for multiple testing with significance at *q* < 0.05.

### *Igf2* enhancer deletion in mice

A 4.9-kb-long DNA fragment (chr7: 149,796,331-149,801,250 in mm9) was deleted from the intergenic region of *H19* and *Igf2* by classical ES cell gene targeting and blastocyst injection in the mouse on the 129S1 genetic background. One *loxP* site remained at the site of the deletion mutation after the excision of the Pgkneo positive selection cassette by crossing the targeted mutant male mouse to an Hprt-CRE transgenic female^80^. Three oligonucleotide primers, IGKOCrerecU: CGGAATGTTTGTGTGGAGAGCA; IGKOwtU: TAGGGGTCCTGAAGACGTCAG; and IGKOCreWTL: TTGGTGTAGCACCCTGTAACCC are combined in one PCR reaction to distinguish the mutant from the wild type allele, as visualized by a 450 bp or a 350 bp long PCR product, respectively. Notably, the enhancer we deleted in mice was the closest enhancer (as defined by ENCODE) to the one we found epigenetic misregulated at the *IGF2* locus in major psychosis patients. We identified mouse enhancer boundaries using chromatin marks in the mouse forebrain (ENCODE; ref. ^2^; Fig. 3) and conservatively deleted a ~4.9-kb intergenic region encompassing the full length of the enhancer.

Mice were bred and housed in ventilated polycarbonate cages, and given *ad libitum* sterile food (LabDiet 5021) and water. Adult mice were housed by sex in groups of 2–5 littermates. The vivarium was maintained under controlled temperature (21 °C±1 °C) and humidity (50–60%), with a 12-h diurnal cycle (lights on: 0700–1900). Approximately equal numbers of male and female were tested, and no sex differences were detected (in all Western blotting, HPLC, and transcriptomic experiments). No animals were excluded from the study. Wild-type (+/+) and homozygous knock-out (*Igf2*enh−/−*)* adult mice (~2.5 months old) were tested, and sample sizes were comparable to other studies of *Igf2* mutant mice^17,81^. All animal procedures were approved by the Institutional Animal Care Committee of the Van Andel Research Institute and complied with the requirements of the Institutional Animal Care and Use Committee (AUP # PIL-17-10-010).

### Chromatin interaction analysis in mice

We analyzed Hi-C data from mouse cortical neurons (accession: GSE96107)^82^. Our Hi-C analysis pipeline involved Trim Galore (v0.4.3) for adapter trimming, HiCUP^83^ (v0.5.9) for mapping and performing quality control, and GOTHiC for identifying significant interactions (Bonferroni *p* < 0.05), with a 40-kb resolution^84^ (R package, v1.16.0). GOTHiC is an effective tool for identifying cis-interactions (interactions at shorter mean distances)^85^. Hi-C gene annotation involved identifying interactions with gene promoters, defined as ±2 kb of a gene transcription start site.

### Immunoblotting

All tissue preparation procedures were performed on ice. Frozen tissue samples weighing ~20 mg were sonicated in 500 µl of RIPA buffer (10 mM Tris-HCl pH 8, 1% Triton X-100, 0.1% sodium deoxycholate, 0.1% SDS, 140 mM NaCl, protease inhibitor cocktail from Roche, and 1 mM EDTA) and incubated for 1 h on ice with mixing. The samples were then centrifuged at 22,000×g for 30 min. Protein content of the supernatant was determined using a BCA assay (Thermo Fisher Scientific) and then diluted in SDS-PAGE sample buffer (Biorad) to yield 20 µg protein per lane. Samples were separated on 4–20% SDS-PAGE gels (Thermo Fisher Scientific) and blotted onto 0.22 µm PVDF membranes (Thermo Fisher Scientific) for 2 h at a constant 20 V using xCell II blot module (Thermo Fisher Scientific). Membranes were blocked in TBST (50 mM Tris-HCl pH 7.6, 150 mM NaCl, and 1% tween-20) containing 5% non-fat dry milk (Bio-Rad) for 1 h at room temperature. Membranes were then incubated with blocking buffer containing primary antibodies: tyrosine hydroxylase (TH, Pel-Freez Biologicals, P40101-150), NeuN (Cell Signaling), internexin neuronal intermediate filament protein (INA, Sigma, HPA008057), and actin (Millipore, MAB1501) diluted 1:1000 overnight at 4 °C. Membranes were washed three times for 5 min with TBST and probed with the appropriate HRP-conjugated anti-IgG antibodies (Cell Signaling, anti-rabbit IgG#7074 and anti-mouse IgG#7076,) diluted 1:6000 in blocking buffer according to the manufacturers’ recommended protocol. Blots were then washed three times for 5 min with TBST and imaged using west pico ECL reagent (Thermo Fisher Scientific).

### HPLC-based quantification of dopamine levels

All tissue preparation procedures were performed on ice. Frozen tissue samples weighing between 5 and 20 mg were sonicated in 100–300 µl of 0.2 M perchloric acid (Sigma). The sample was centrifuged at 22,000×*g* for 30 min and the resulting supernatant was filtered using 0.22 µM cellulose acetate filter (Costar). The filtered supernatant was separated using the HTEC-500 High Pressure Liquid Chromatography (HPLC) system (Eicom) with the SC-30DS reverse phase separation column (Eicom) and electrochemical detector. Samples were separated in mobile phase consisting of 0.1 M citrate acetate pH 3.5, 20% methanol, 220 g/L sodium octane sulfonate, and 5 mg/L EDTA-Na. The samples were then compared to known standards of dopamine (Sigma), homovinillic acid (HVA), and 3,4-Dihydroxyphenylacetic acid (DOPAC). The pellet was dissolved in 0.5 mL of 1 M NaOH for 10 min at 90 °C, and the resulting protein concentration determined by BCA assay. The final values were calculated as ng analyte per µg protein.

### RNA-seq processing for mice with the *Igf2* enhancer deletion

A transcriptomic analysis of the striatum and frontal cortex of wild-type and *Igf2*enh−/− mice was performed. Brain tissue (~25 mg) was homogenized with a ceramic bead-based homogenizer (Precellys, Bertin Instruments) in 1 mL of Trizol (Life Technologies). Total RNA was isolated according to the Trizol manufacturer’s instructions, treated with RNase-free DNase I (Qiagen) at room temperature for 30 min, and cleaned up with the RNeasy Mini Kit (Qiagen). RNA yield was quantified using a NanoDrop ND-1000 (Thermo Fisher Scientific), and RNA integrity was verified via the Agilent Bioanalyzer 2100 system (Agilent Technologies). Libraries were prepared by the Van Andel Genomics Core from 500 ng of total RNA and sequenced, as described in the Gene expression profiling by RNA-seq Methods section above.

Single-end 75 bp reads were generated for the RNA-seq experiment. Trimgalore v0.5.0 (https://www.bioinformatics.babraham.ac.uk/projects/trim_galore/) was used to trim low-quality bases. STAR^66^ was used to align the reads to the genome. The genome index was generated using STAR using GRCm38.primary_assembly.genome.fa as the reference sequence and gencode.vM15.primary_assembly.annotation.gtf as the gene definition file. The genome fasta sequence was downloaded from ftp://ftp.sanger.ac.uk/pub/gencode/Gencode_mouse/release_M15/GRCm38.primary_assembly.genome.fa.gz and gene definitions from ftp://ftp.sanger.ac.uk/pub/gencode/Gencode_mouse/release_M15/gencode.vM15.primary_assembly.annotation.gtf.gz.

### Differential expression and pathway analysis in mice

Only genes with ≥1CPM in all samples were included for differential expression analysis. Transcript counts were normalized for library size using the Trimmed Mean of M values (TMM) method. Ensembl Gene IDs were mapped to MGI symbols using Biomart^86^. To ascertain differentially-expressed genes, edgeR^34^ was used to fit a linear model to each gene, using genotype (wild-type or *Igf2*enh−/−) as an explanatory variable and sex as a covariate. estimateDisp() was used to estimate the dispersion of each gene, and glmLRT was used to identify differentially-expressed genes. Benjamini-Hochberg was used to correct for multiple-testing (significance at *q* < 0.05).

For pathway analysis, pre-ranked GSEA was run using the output of differential expression analysis^35^ (1000 permutations). Gene sets included those from curated pathway databases including: HumanCyc^72^, IOB’s NetPath^73^, Reactome^74,75^, NCI Curated Pathways^76^, Pathway Interaction database, MSigDB^35^, Panther^77^, and Gene Ontology Biological Pathway terms (no iea)^78,79^, downloaded from http://download.baderlab.org/EM_Genesets/October_01_2017/Mouse/symbol/Mouse_GOBP_AllPathways_no_GO_iea_October_01_2017_symbol.gmt;^87^. Gene sets were limited to those with 10–200 genes (6321 gene sets).

### Synaptosomal proteome analysis by mass spectrometry

Changes in synaptic proteins in the striatum of mice with the enhancer deletion at *Igf2* was determined by quantitative proteome analysis. In this study, striatal synaptosomes from wild-type and *Igf2*enh−/− mice were compared (2 striatum pools per genotype, 3 mice per pool; *n* = 6 wild-type and 6 *Igf2*enh−/− mice). Synaptosomes were isolated from frozen striatum similar to a previously-described protocol^88^. Specifically, striatum tissue was homogenized in 5 mL isolation buffer (0.32 M sucrose, 10 mM Hepes pH 8.0, and protease inhibitor cocktail) using 16 gentle strokes with a glass dounce homogenizer. Samples were then centrifuged for 10 min at 1000×*g*. The resultant supernatant was then layered on 1.2 M sucrose and centrifuged at 160,000×*g* for 15 min using SW-41-Ti rotor (Beckman Coulter). The interface between sucrose layers was collected, layered on top of 0.8 M sucrose, and centrifuged again at 160,000×*g* for 15 min. The resulting pellet was then dissolved in 100 μl RIPA buffer and concentration determined using a BCA assay (Thermo Fisher Scientific). Purity of synaptosomes was verified by western blotting with anti-synaptophysin antibody (Cell Signaling, #12270), anti-histone 3 antibody (Abcam, ab1791), and anti-actin antibody (Millipore, #MAB1501) all diluted 1:1000. Each sample (70 µg per sample) were run 1 cm into a SDS-PAGE gel and stained using coomassie blue as described^89^.

Samples were then submitted to the Whitehead Mass Spectrometry Facility (MIT, Cambridge, MA) for subsequent proteome library preparation, iTRAQ-labeling, chromatographic separation, and mass spectrometry (MS). Briefly, samples excised from the SDS-PAGE gel were reduced, alkylated, and digested with trypsin at 37 °C overnight using buffers and reagents that were free of primary amines. The resulting peptides were extracted, labeled with Sciex iTRAQ 4-plex isotopic tags, combined, purified, and concentrated by solid-phase extraction and injected onto a Shimadzu HPLC and fraction collector equipped with a self-packed Aeris PEPTIDE XB-C18 analytical column (10 cm by 2.1 mm, Phenomenex). Peptides were eluted using standard reverse-phase gradients and pH = 10 ammonium formate buffers with a total of 16 fractions collected across the analytical gradient. The resulting fraction were reduced to a total of 8 fractions. After volume reduction the peptides in these eluents were separated using standard reverse-phase gradients using a Thermo EASY nLC chromatographic system. The effluent from the column was analyzed using a Thermo Q Exactive HF-X Hybrid Quadrupole-Orbitrap mass spectrometer (nanospray configuration) operated in a data-dependent manner.

Peptides were identified from the MS data using PEAKS Studio 8.5. The *Mus musculus* Refseq protein FASTA entries were downloaded from NIH/NCBI and concatenated to a database of common contaminants. An FDR threshold of 1% for identification of peptides and protein positive identifications was used, and quantitation was based on the top three Total Ion Current (TIC) method. Relative ratios of the iTRAQ 4-plex reporter ions were used for quantitation. Significance was calculated by ANOVA, and the Benjamini-Hochberg method was used for multiple testing correction (significance *q* < 0.05). As a check for purity of synaptosomal protein content, we performed a pathway analysis on all detected proteins. A hypergeometric test was performed using pathway genes (10–200 genes) and human-mouse disease genes (total of 1760 test). Foreground was all proteins detected (1098 unique MGI symbols), and background was all proteins in all pathways (9593 MGI symbols). Pathways inclusion required ≥1 genes in the foreground and ≥1 genes in the background set (1422 pathways). Benjamini-Hochberg correction was used for multiple testing correction. Three hundred and twenty pathways were significantly enriched (*q* < 0.05). Main pathway themes corresponded to synaptic neurotransmission, with minor themes corresponding to glucose metabolism and immune activity and signaling (Supplementary Fig. 17, Supplementary Data 19).

To identify pathways of proteins differentially enriched in *Igf2*enh−/− mice relative to wildtype, pathway analysis was performed using MetaCore (https://clarivate.com/products/metacore/). Proteins with differential expression at *p* < 0.001 (*q* < 0.01) were used as foreground, and the set of all proteins for which relative ratio was computed was used as the background.

### Software availability

Software used to produce the results in this work are publicly available in a github repository at https://github.com/shraddhapai/EpiPsychosis_IGF2.

### Reporting summary

Further information on research design is available in the Nature Research Reporting Summary linked to this article.

## Supplementary information

Supplementary Information

Description of Additional Supplementary Files

Supplementary Data 1

Supplementary Data 2

Supplementary Data 3

Supplementary Data 4

Supplementary Data 5

Supplementary Data 6

Supplementary Data 7

Supplementary Data 8

Supplementary Data 9

Supplementary Data 10

Supplementary Data 11

Supplementary Data 12

Supplementary Data 13

Supplementary Data 14

Supplementary Data 15

Supplementary Data 16

Supplementary Data 17

Supplementary Data 18

Supplementary Data 19

Reporting Summary

Source Data

## Data Availability

Raw and processed data for data generated in this work have been deposited at the Gene Expression Omnibus under the SuperSeries accession number GSE112525. These include subseries for human DNA methylation arrays (GSE112179), RNA-sequencing (GSE112523), bisulfite targeted sequencing (GSE112524), and genotyping arrays (GSE113093), and transcriptome profiling of mouse brains (GSE120423). These data are associated with Figs. 1, 2, and 4 and Supplementary Figs. 3–9, 14 and 15. The chromatin conformation analysis in human prefrontal cortex, as shown in Fig. 3a and Supplementary Fig. 10, used peaks provided from the 3D Interaction Database at https://www.kobic.kr/3div/. Protein-protein interaction networks shown in Fig. 4c and Supplementary Fig. 7c were obtained from the STRING database (https://string-db.org/). The mass spectrometry proteomics data have been deposited to the ProteomeXchange Consortium via the PRIDE partner repository (https://www.ebi.ac.uk/pride/archive/); dataset identifiers are PXD012786 and 10.6019/PXD012786. The underlying data for Fig. 3b–e and Supplementary Figs. 12 and 13 are available in the Source Data file. All other relevant data supporting the key findings of this study are available within the article and its Supplementary Information files or from the corresponding authors upon reasonable request. A reporting summary for this Article is available as a Supplementary Information file.
