# Supplementary material for: Differential methylation of enhancer at IGF2 is associated with abnormal dopamine synthesis in major psychosis
Source: Nat Commun. 2019 May 3;10:2046. doi: 10.1038/s41467-019-09786-7 (PMC6499808; doi:10.1038/s41467-019-09786-7)
Supplement: Supplementary file 1 — Supplementary Information [file 41467_2019_9786_MOESM1_ESM.doc]

# Supplementary Information for

# **Differential methylation of enhancer at *IGF2* is associated with abnormal dopamine synthesis in major psychosis**

Pai *et al.*

Corresponding author: Viviane Labrie (viviane.labrie@vai.org)

**This PDF file includes:**

Supplementary Note 1

Supplementary Figures 1-17

Supplementary Table 1

**Other Supplementary Material for this manuscript includes the following:**

Supplementary Data 1. Patient information

Supplementary Data 2. Comb-p results for DMR calls in EPIC microarrays

Supplementary Data 3. Pathway analysis of EPIC array probes

Supplementary Data 4. Summary statistics for RNA sequencing

Supplementary Data 5. Differential expression results for case-control RNAseq data

Supplementary Data 6. Pathway analysis for transcriptomic data from GSEA

Supplementary Data 7. Results of cis meQTL analysis of DMRs from the EPIC arrays

Supplementary Data 8. Results of meQTL analysis of EPIC DMRs with SCZ GWAS hits and credible SNPs

Supplementary Data 9. Genotype-disease interaction of cis-SNPs in linkage equilibrium in IGF2 DMR region

Supplementary Data 10. Summary statistics for processing pipeline of targeted bisulfite sequencing (SeqCapEPI)

Supplementary Data 11. RNA-seq of wild-type and *Igf2*enh-/- mice

Supplementary Data 12. Differential expression in *Igf2*enh-/- mice in frontal cortex

Supplementary Data 13. Differential expression in *Igf2*enh-/- mice in striatum

Supplementary Data 14. Pathway enrichment results for *Igf2*enh-/- mice for frontal cortex

Supplementary Data 15. Pathway enrichment results for *Igf2*enh-/- mice for striatum

Supplementary Data 16. Enriched synaptosomal proteins in striatum of *Igf2*enh-/- mice, compared to wild-type mice. Ratio values above 1 are higher in the wildtype; those under 1 are higher in *Igf2*enh-/- mice

Supplementary Data 17. Disease pathways altered in synaptosomes of *Igf2*enh-/- relative to wild-type mice

Supplementary Data 18. Biological process pathways altered in synaptosomes of *Igf2*enh-/- mice relative to wild-type mice

Supplementary Data 19. Mouse synaptosome enrichment analysis (Biological processes)

# Supplementary Notes

## Supplementary Note 1: Software Used for Analysis

| **Name** | **Ver.** | **URL** | **Ref** |
| --- | --- | --- | --- |
| BSMap | 2.74 | <https://code.google.com/archive/p/bsmap/downloads> | 1 |
| Picard | 2.9.4-SNAPSHOT | <https://github.com/broadinstitute/picard>  http://picard.sourceforge.net |  |
| Bedtools | 2.26 | https://github.com/arq5x/bedtools2 | 2 |
| Samtools | 1.5 | <http://www.htslib.org/> | 3 |
| FastQC | v0.11.5 | <https://www.bioinformatics.babraham.ac.uk/projects/fastqc/> |  |
| Trimmomatic | 0.36 | <http://www.usadellab.org/cms/?page=trimmomatic> | 4 |
| Tabix (HTSutils) | 1.5 | <http://www.htslib.org/> | 5 |
| edgeR | 3.18.1 | <http://bioconductor.org/packages/release/bioc/html/edgeR.html> | 6 |
| Trimgalore | 0.5.0 | https://www.bioinformatics.babraham.ac.uk/projects/trim_galore/ |  |
| STAR | 2.5.3a | https://github.com/alexdobin/STAR | 7 |
| Minfi | 1.22.1 | <http://bioconductor.org/packages/release/bioc/html/minfi.htmlcyr> | 8 |
| Cytoscape | 3.5.1 | http://cytoscape.org/ | 9 |
| GSEA | 3.0 | http://software.broadinstitute.org/gsea/index.jsp | 10 |
| EnrichmentMap | 3.1.0RC4 | http://apps.cytoscape.org/apps/enrichmentmap | 11 |
| AutoAnnotate | 1.2 | http://apps.cytoscape.org/apps/autoannotate | 12 |
| CIBERSORT |  | [http://cibersort.stanford.edu](http://cibersort.stanford.edu/) | 13 |
| Plink | 1.90b4.9 | https://www.cog-genomics.org/plink/1.9/ | 14 |
| Michigan Imputation Server |  | <https://imputationserver.sph.umich.edu/index.html> | 15 |
| Eagle | 2.3 | (part of Michigan Imputation Server) | 16 |
| Check-Bim |  | http://www.well.ox.ac.uk/~wrayner/tools/#Checking |  |
| Pathway databases |  | [http://humancyc.org](http://humancyc.org/)  [http://www.netpath.org](http://www.netpath.org/)  [http://www.reactome.org](http://www.reactome.org/)  <http://software.broadinstitute.org/gsea/msigdb/>  <http://pantherdb.org/>  http://download.baderlab.org/EM_Genesets/October_01_2017/Human/symbol/ Human_GOBP_AllPathways_no_GO_iea_October_01_2017_symbol.gmt  Mouse: <http://download.baderlab.org/EM_Genesets/October_01_2017/Mouse/symbol/Mouse_GOBP_AllPathways_no_GO_iea_October_01_2017_symbol.gmt>  <https://clarivate.com/products/metacore/> | 10, 11, 17-22 |
| String |  | <https://string-db.org/> | 23 |
| GATK | 3.8.0 | https://software.broadinstitute.org/gatk/ | 24 |
| SciNet |  | https://www.scinethpc.ca/ | 25 |

# Supplementary Figures


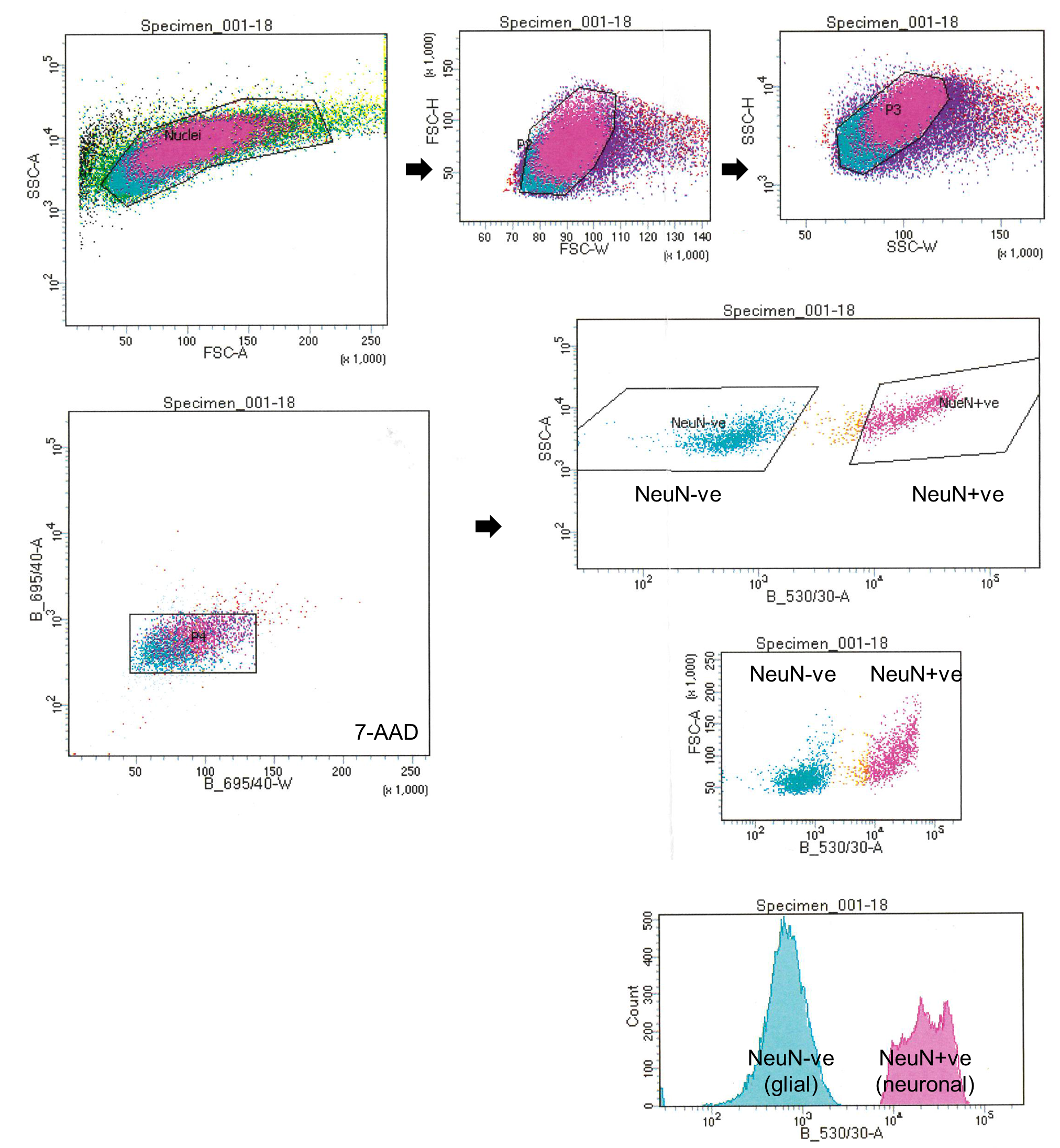


**Supplementary Figure 1.**  Isolation of human prefrontal cortex neurons by flow cytometry. Representative gating image for flow cytometry sorting of neuronal (NeuN+) and glial/non-neuronal (NeuN-) nuclei, after gating for single nuclei and 7-AAD positive nuclei. Reanalysis of NeuN+ aliquots after sorting show a high purity for NeuN+ stained nuclei (on average 96%).


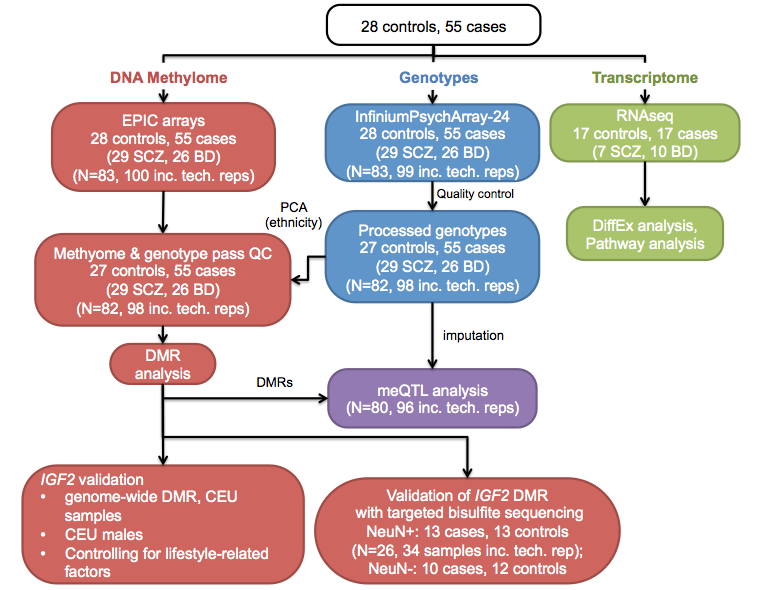


**Supplementary Figure 2.** Experimental design for genomic profiles and key analyses. The flowchart shows the sample count and workflow for key analyses for the DNA methylome (red), genotype (blue) and transcriptomic (green) data generated in this work. Samples selected for transcriptome profiling were a subset of those used for genome-wide DNA methylation profiling.


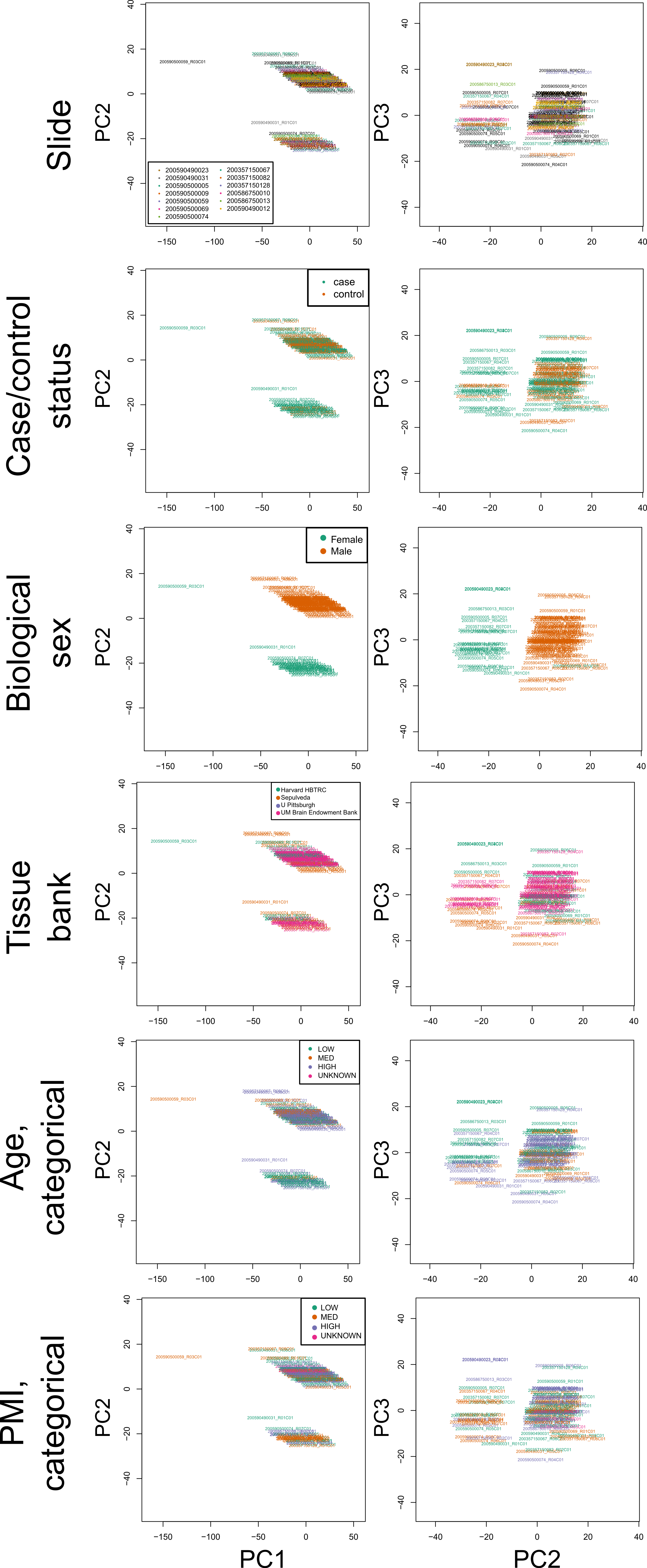


**Supplementary Figure 3.** Principal component projections of DNA methylation (normalized beta values) from EPIC microarrays, with samples colour-coded by various biological and technical replicates. Each row shows projections of PC2 vs PC1 (left) and PC3 vs PC2 (right) for a given variable. One outlier sample is visible in all plots; this one was excluded from further analyses. Images show that the samples cluster by sex; sex was included as a covariate in the DNA methylation analysis.


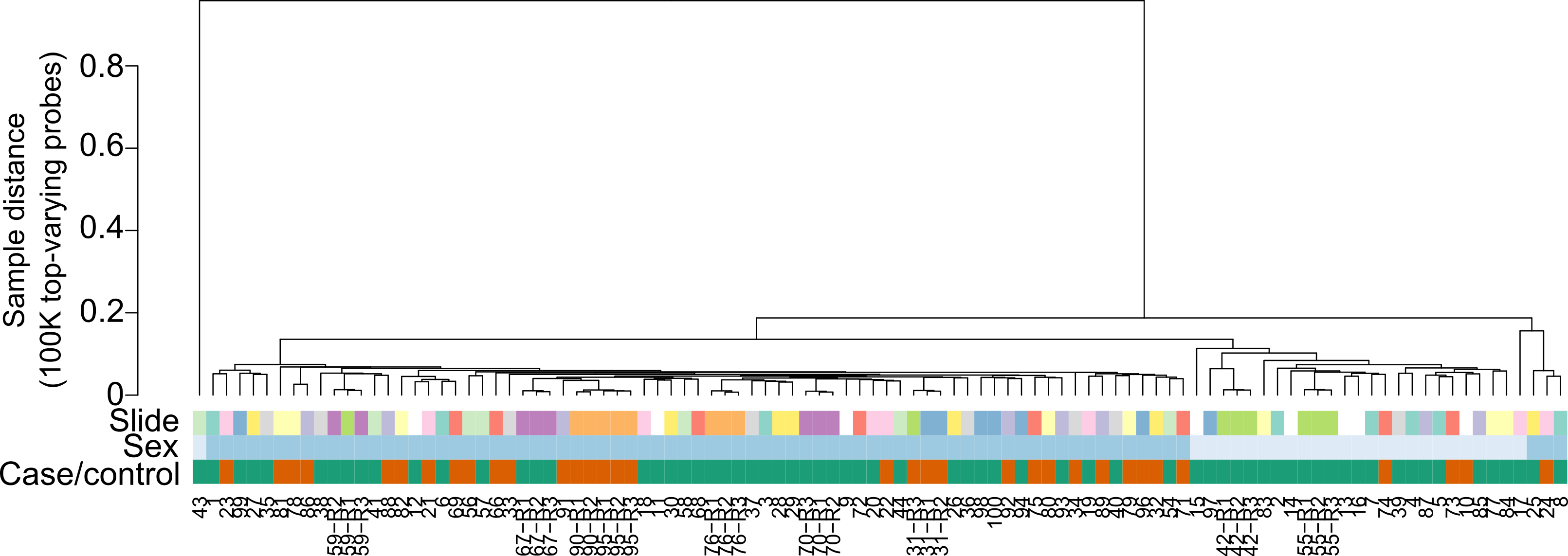


**Supplementary Figure 4.** Hierarchical clustering of MethylationEPIC array samples using 100K probes with highest variance. Samples are coded by microarray slide, sex, and case/control status. Sample suffixed with –R1,2,3 are technical replicates. All samples are NeuN+ except for one NeuN- sample at the left branch of plot.


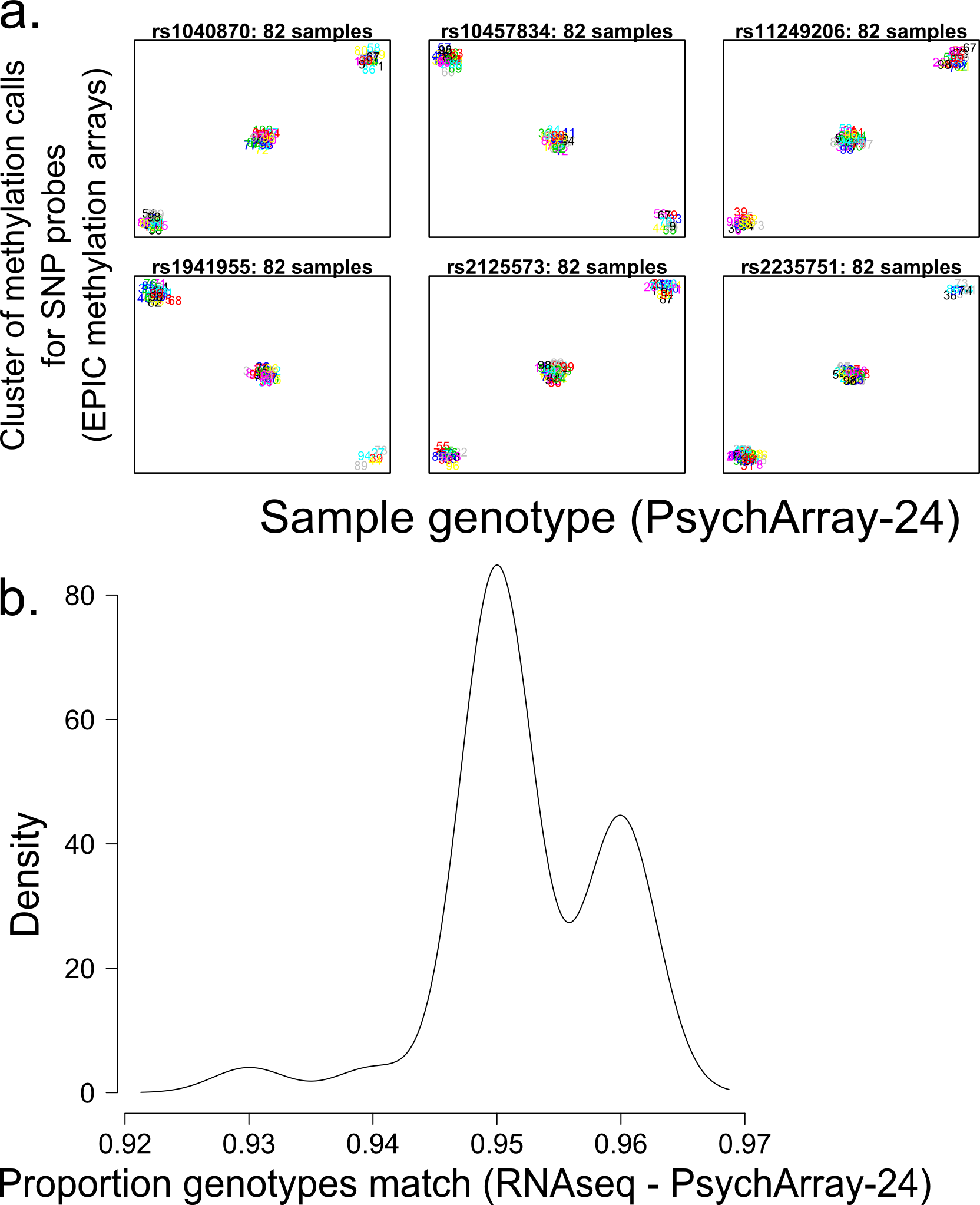


**Supplementary Figure 5:** Verification of data match in DNA methylation, transcriptomic, and SNP array analysis. (**a**) Correlation of genotypes as measured by the SNP arrays (x-axis) and inferred from EPIC arrays (y-axis). Data shown for representative SNPs in common with the EPIC microarray SNP probes and those measured by the Infinium PsychArray-24. Genotypes were inferred from EPIC SNP probes by fitting a 3-component mixture model to SNP beta values using mclust (<https://github.com/ttriche/infiniumSnps>). Images demonstrate that DNA methylation and genotype was accurately matched for each sample. The panel shows data for 6 SNPs, but results were identical for 13 SNPs tested. (**b**) Percent overlap in per-sample genotype calls between RNAseq and genotyping platforms (n=34 samples tested; 4.1K-11.8K SNPs, median of 5,202 SNPs).


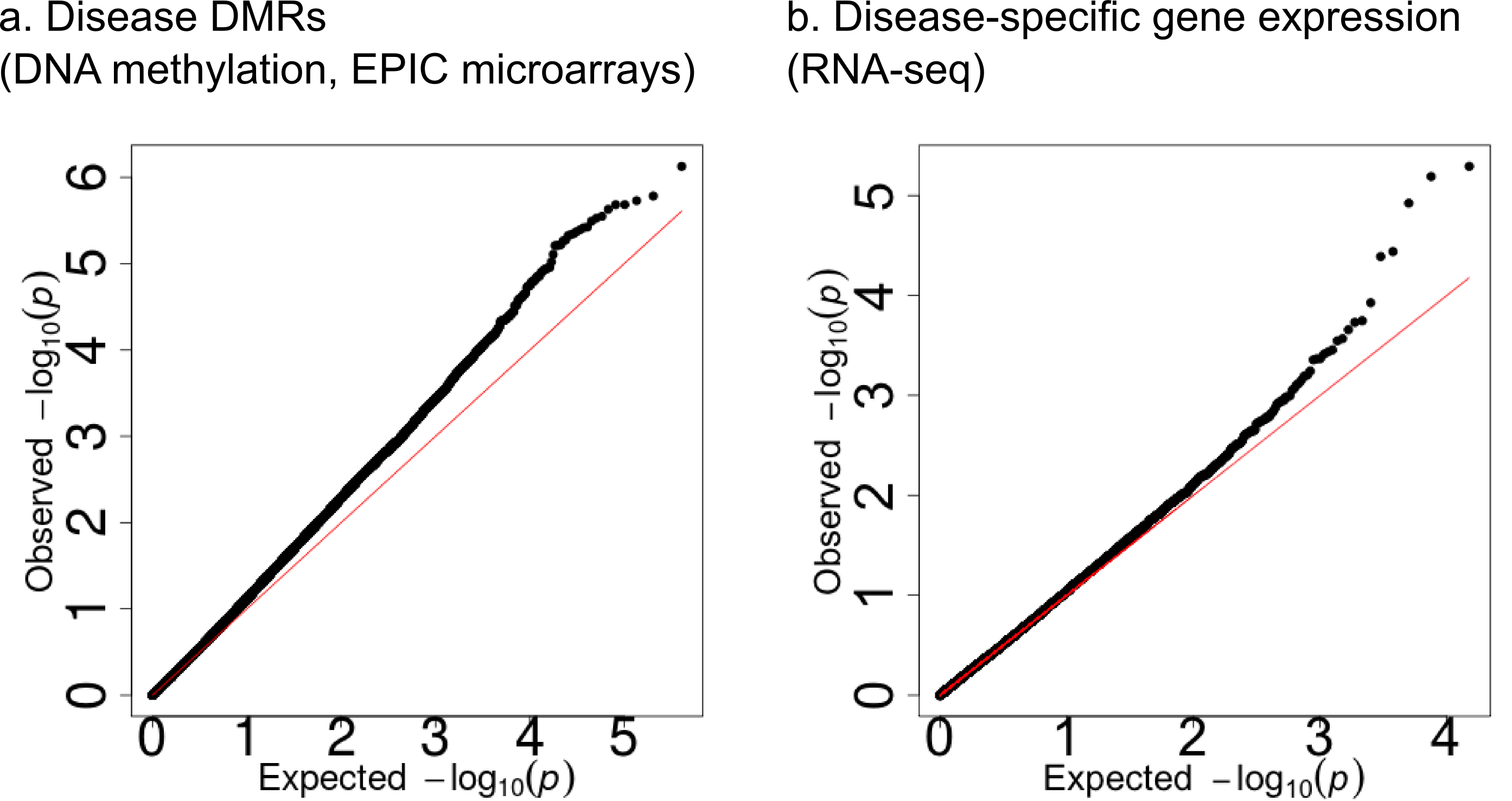


**Supplementary Figure 6.** QQ-plots for DNA methylation region analysis (lambda=1.03, computed using bacon package^26^) (**a**) and RNA-seq analysis (**b**). Plot show comparison between observed and expected differentially methylated regions (DMRs) and gene expression in major psychosis relative to controls. Volcano plots showing an alternate view of probe-level signal are shown in Fig. 1a (DNA methylation) and Supplementary Figure 7a (RNA-seq).


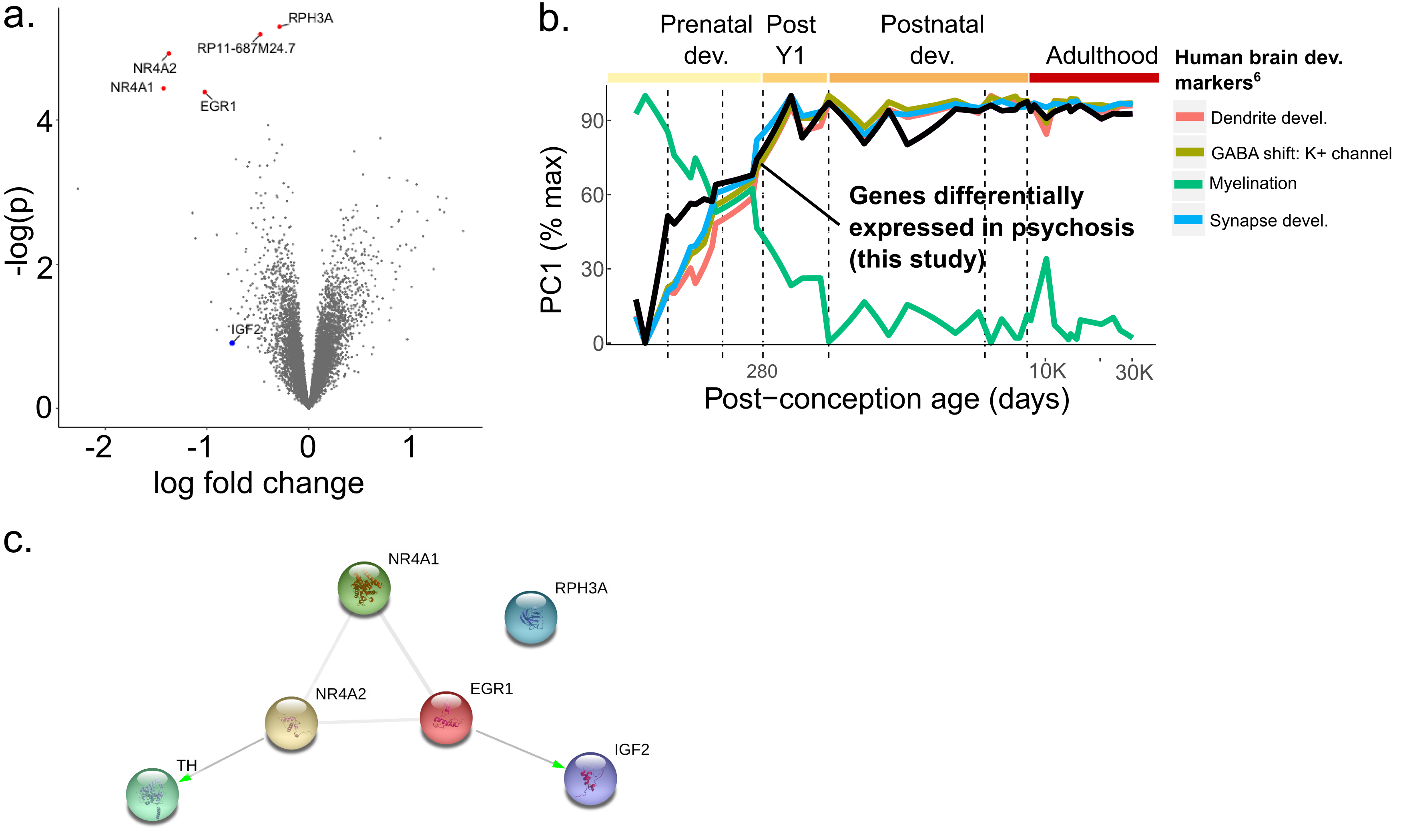


**Supplementary Figure 7.** Transcriptional changes in major psychosis identified by RNA-sequencing. (**a**) Volcano plot of differential expression analysis between major psychosis cases (n=17) and controls (n=17), after controlling for age, sex, post-mortem interval, and neuronal cell percentage. Dots in red have *p*<10^-4^ (generalized linear regression, edgeR^6^), and *IGF2* is highlighted in blue. (**b**) Transcript level changes in human frontal cortex across the human life span, for genes that were differentially expressed in major psychosis. Data from BrainSpan, which included the human frontal cortex and areas of the ganglionic eminence. Genes associated with a developmental process were obtained from BrainSpan (Supplementary Table 13 of that work). Each trendline shows the first principal component of the gene set, scaled from 0-100 across the lifespan. The black trendline represents the genes differentially expressed in the current study (*p*<0.05 generalized linear regression, edgeR, n=854 genes). (**c**) Top differentially expressed genes in major psychosis have known interactions with *IGF2* and *TH* (image from STRING database).

**
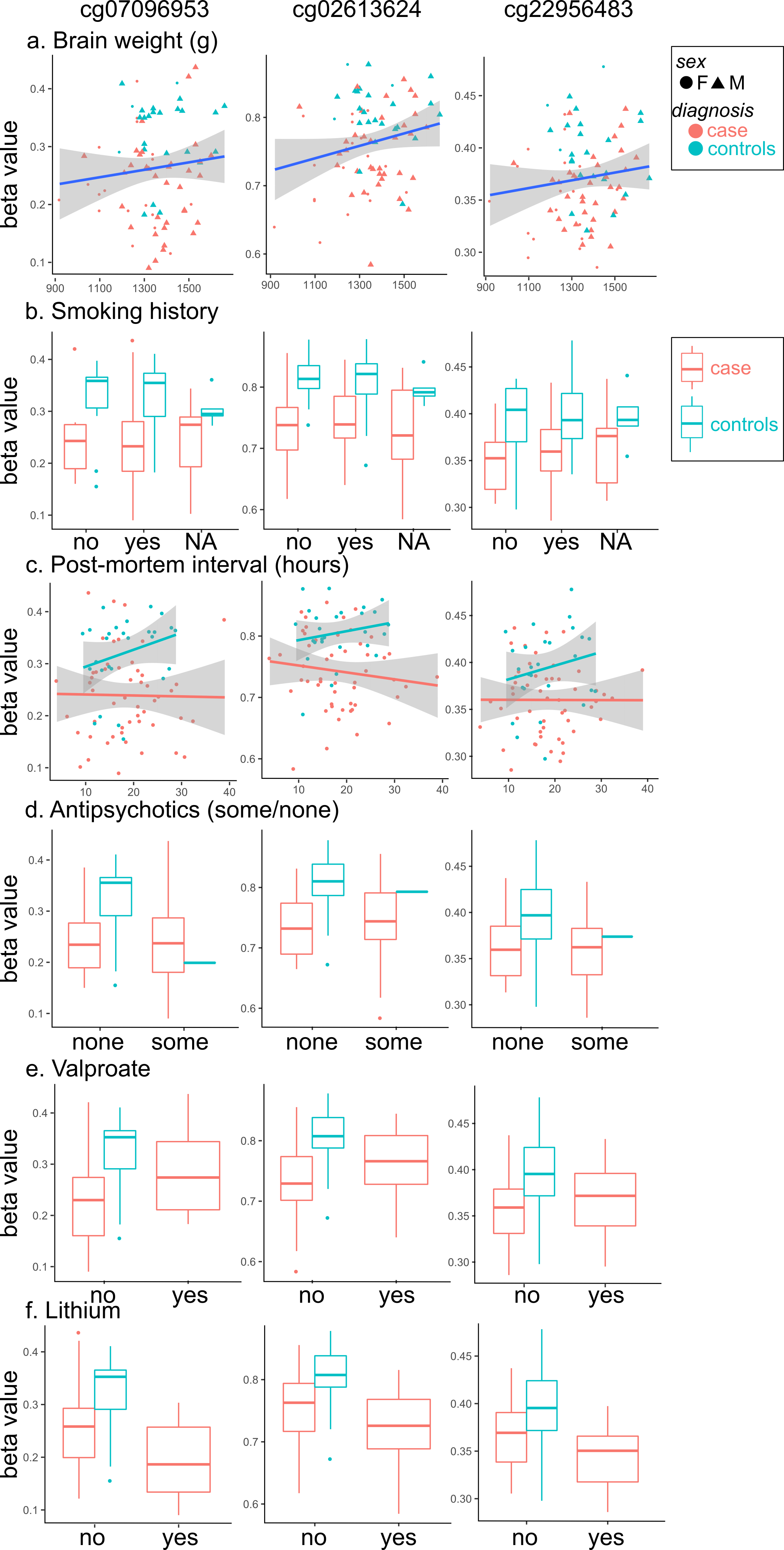
**

**Supplementary Figure 8.** *IGF2* enhancer methylation considering lifestyle factors. Each column shows data for one of the three top probes at the *IGF2* locus differentially methylated in major psychosis. Rows show the effect of brain weight (**a**), smoking (**b**), post-mortem interval (**c**), antipsychotics use (**d**), valproate use (**e**), and lithium use (**f**). Y-axis shows probe-level beta-values. Controls are shown in blue and cases are shown in red. Note that each of these plots shows the marginal DNA methylation (i.e., DNA methylation relative to a single variable, and not accounting for the joint effect of all covariates), whereas the manuscript reports a statistical significance for a nested ANOVA model that jointly considers the effect of all covariates.


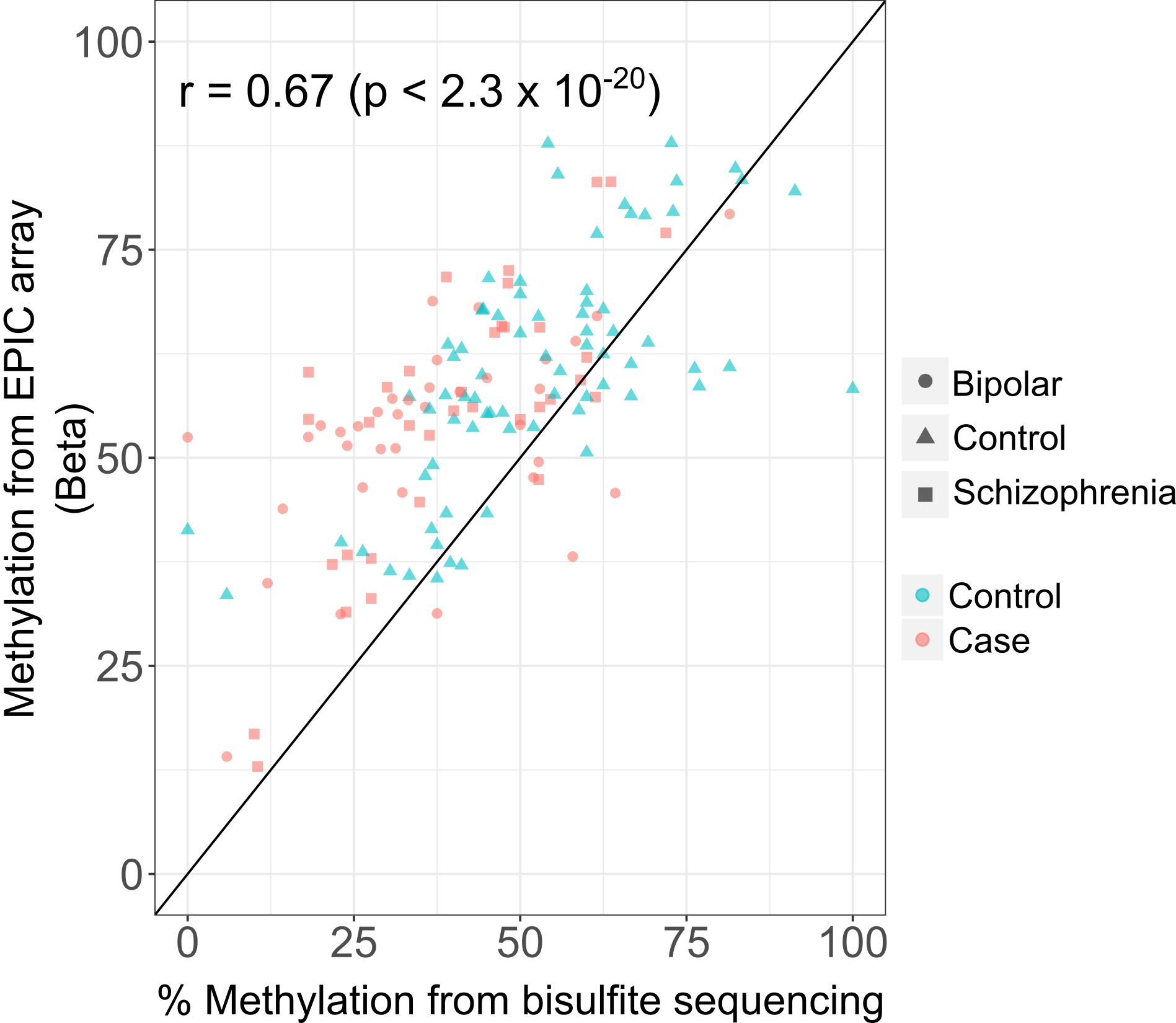


**Supplementary Figure 9.** DNA methylation at the *IGF2* enhancer site, as measured by the EPIC microarray and targeted bisulfite sequencing platforms. Each dot shows base-level methylation for one sample (n=13 controls, 6 bipolar disorder, 7 schizophrenia); only bases with ≥10× coverage for bisulfite sequencing are included (144 data points). X-axis shows DNA methylation measured by bisulfite sequencing, and y-axis shows DNA methylation from the EPIC microarray. P-value for Pearson’s correlation computed by t-test.

**
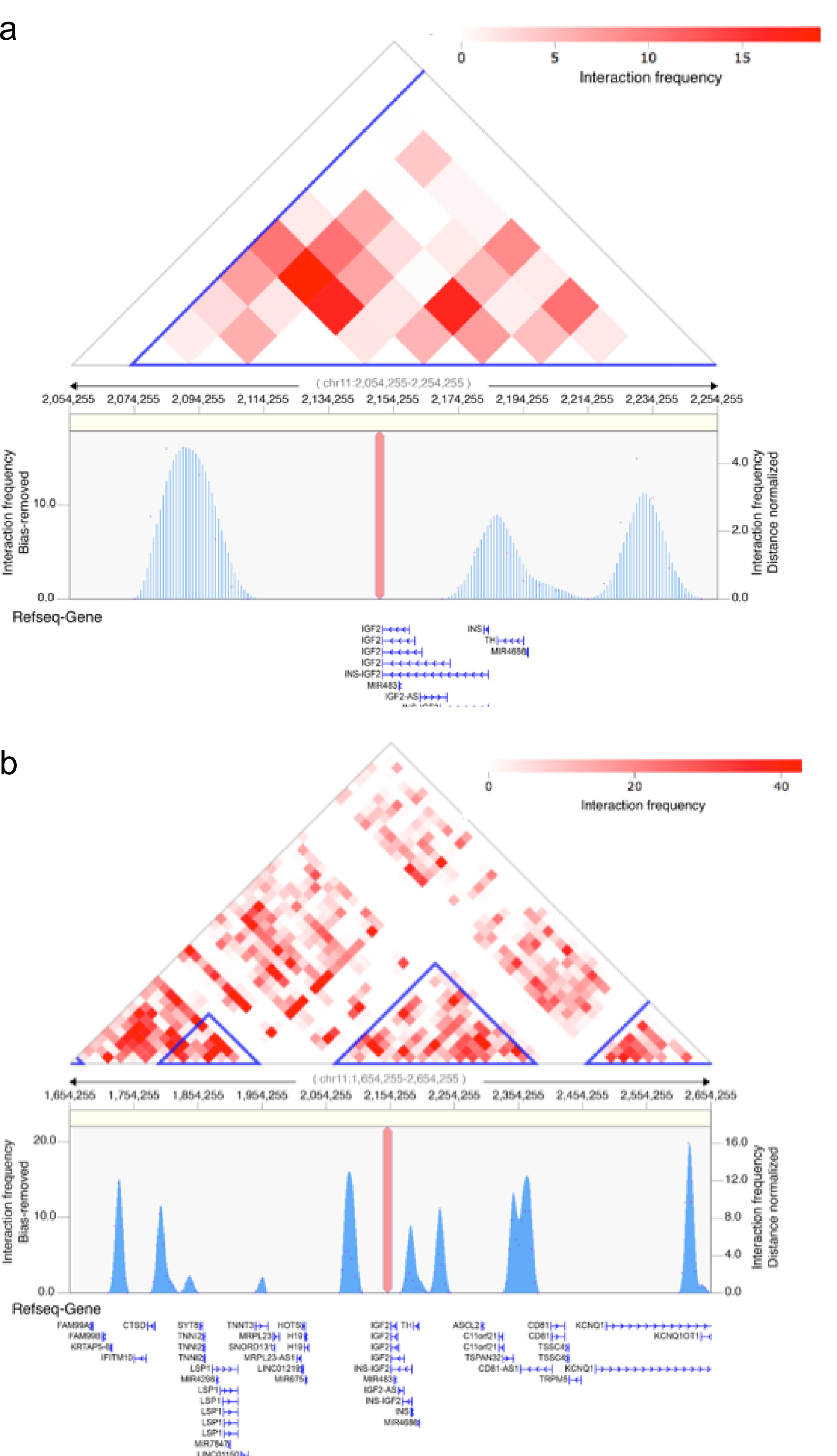
**

**Supplementary Figure 10.** The enhancer at *IGF2* interacts with the *TH* gene. Analysis of chromatin interactions in the human prefrontal cortex at the *IGF2* locus. Interaction ranges are 100 kb (**a**) and 500 kb (**b**) and were obtained from 3D Interaction Viewer and Database (3DIV). In the interaction heat map, topologically associating domains (TAD) are indicated by blue triangles; heat map resolution is 20 kb. Beneath, the one-to-all interaction frequency graph shows the interactions of the enhancer found differentially methylated in major psychosis. The blue bars represent the bias-removed chromatin interaction frequency, and the magenta dots represent distance-normalized interaction frequency. RefSeq Genes are shown.


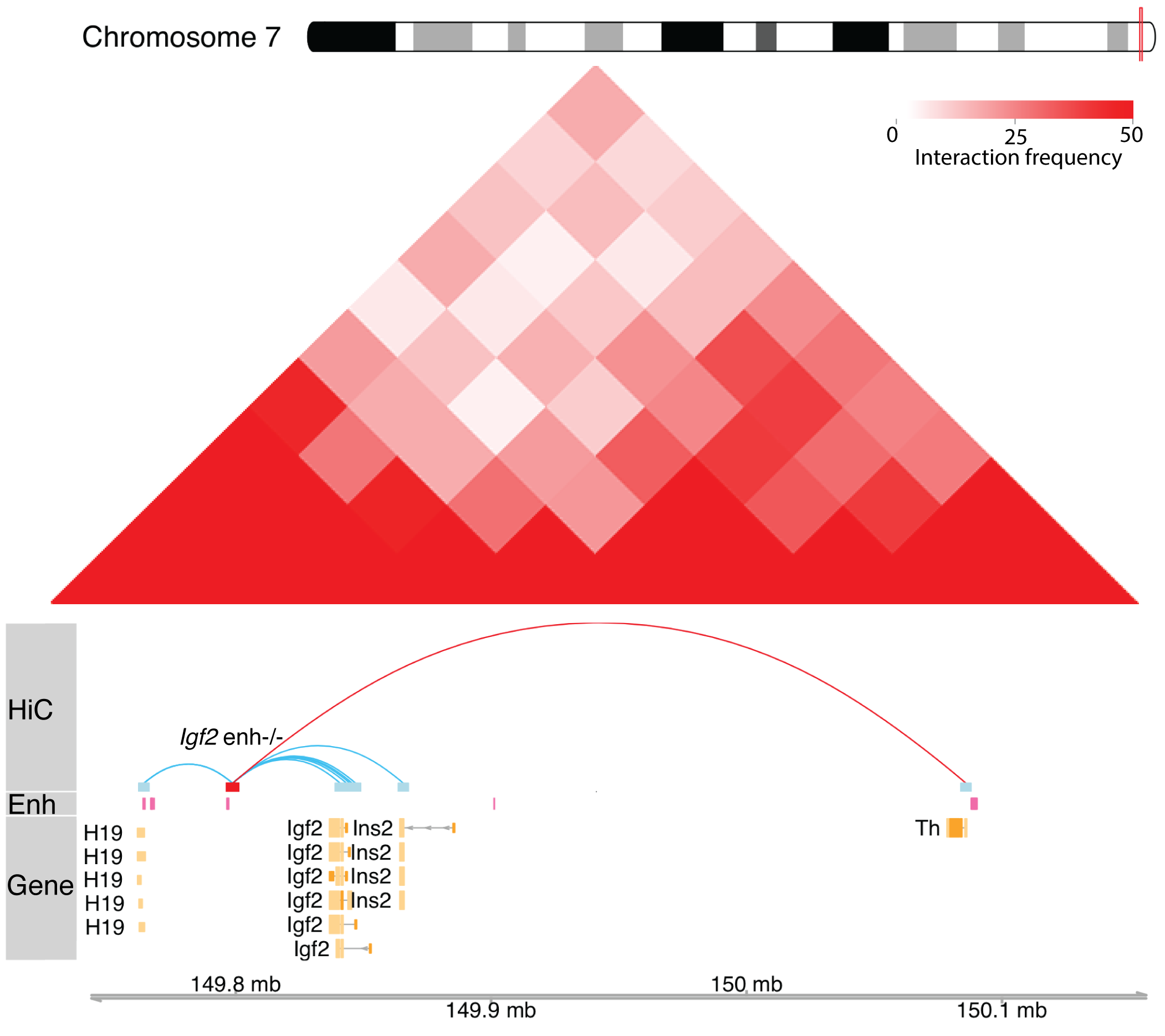


**Supplementary Figure 11.** Chromatin interactions of *Igf2* deleted enhancer in mouse cortical neurons. Heat map of interactions frequencies at a resolution of 40 kb. *Igf2* deleted enhancer targets the promoters of *Th* (shown in red line) as well as other genes (blue lines). Interactions at chr7:149,750,000-150,150,000 shown. Deleted enhancer at *Igf2* is shown (red bar). Mouse forebrain enhancers are shown (ENCODE, pink track).

**
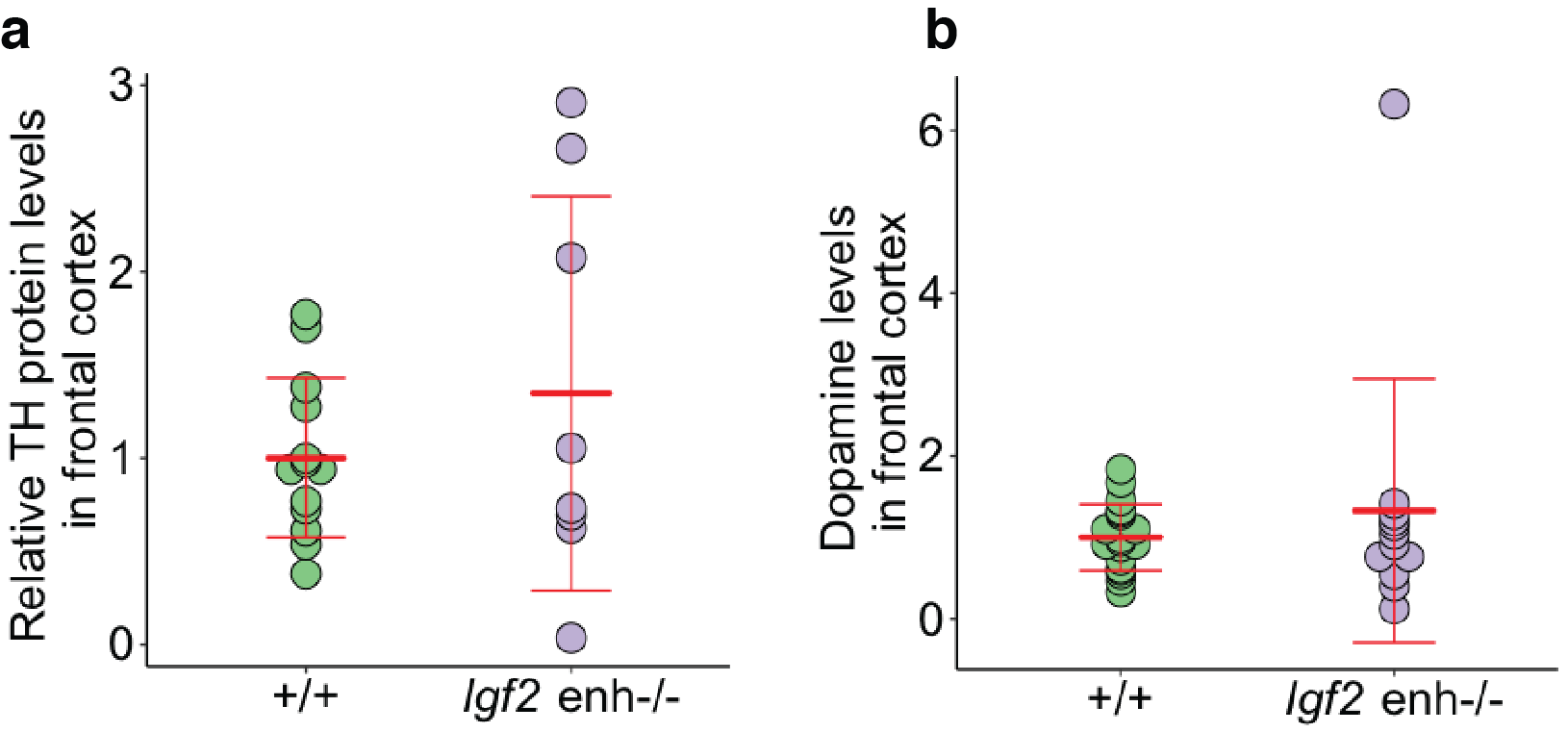
**

**Supplementary Figure 12.** TH protein and dopamine levels are not altered in the frontal cortex of mice with an *Igf2* enhancer deletion. (**a**) Immunoblotting was used to measure TH protein levels in the frontal cortex of adult *Igf2*enh-/- (n=8) and wild-type (+/+; n=13) mice. TH protein levels are relative to control proteins (NeuN, actin). (**b**) Dopamine levels in the frontal cortex of adult *Igf2*enh-/- (n=12) and wild-type (n=19) mice were measured by HPLC. Data normalized to wild-type levels. Data points shown along with mean ± standard deviation.

**
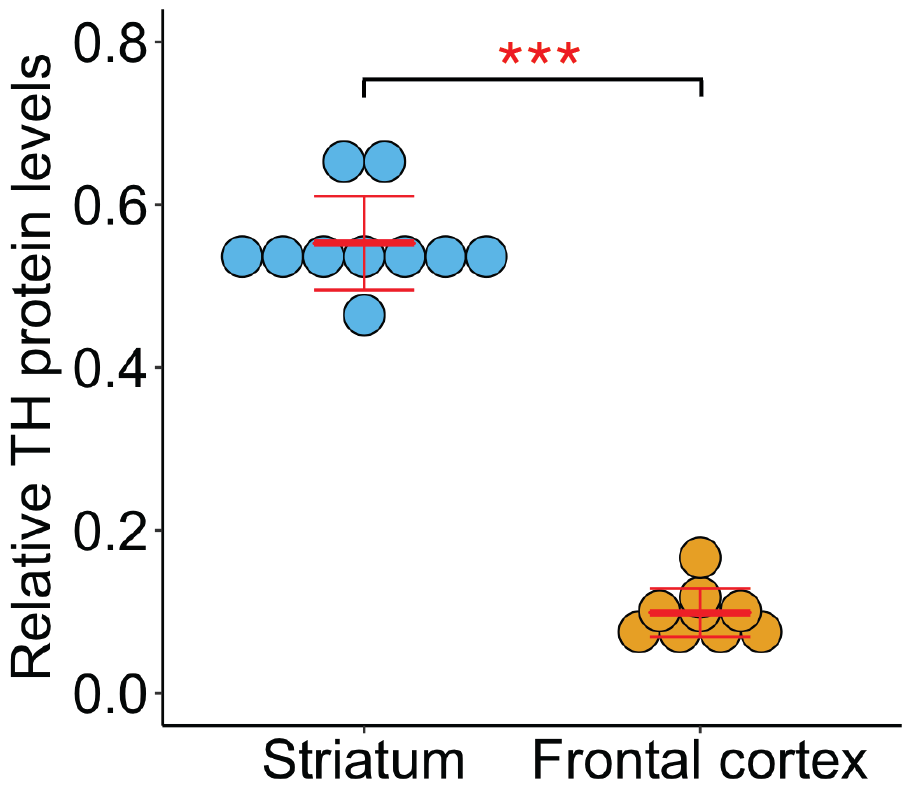
**

**Supplementary Figure 13.** TH protein levels are substantially higher in the striatum compared to the frontal cortex. Comparison of TH protein levels in the striatum (n=10) and frontal cortex (n=9) of adult mice on the 129S1 strain. TH protein levels are relative to control protein (actin). ****p*<10^-11^; one-way ANOVA. Data points shown along with mean ± standard deviation.


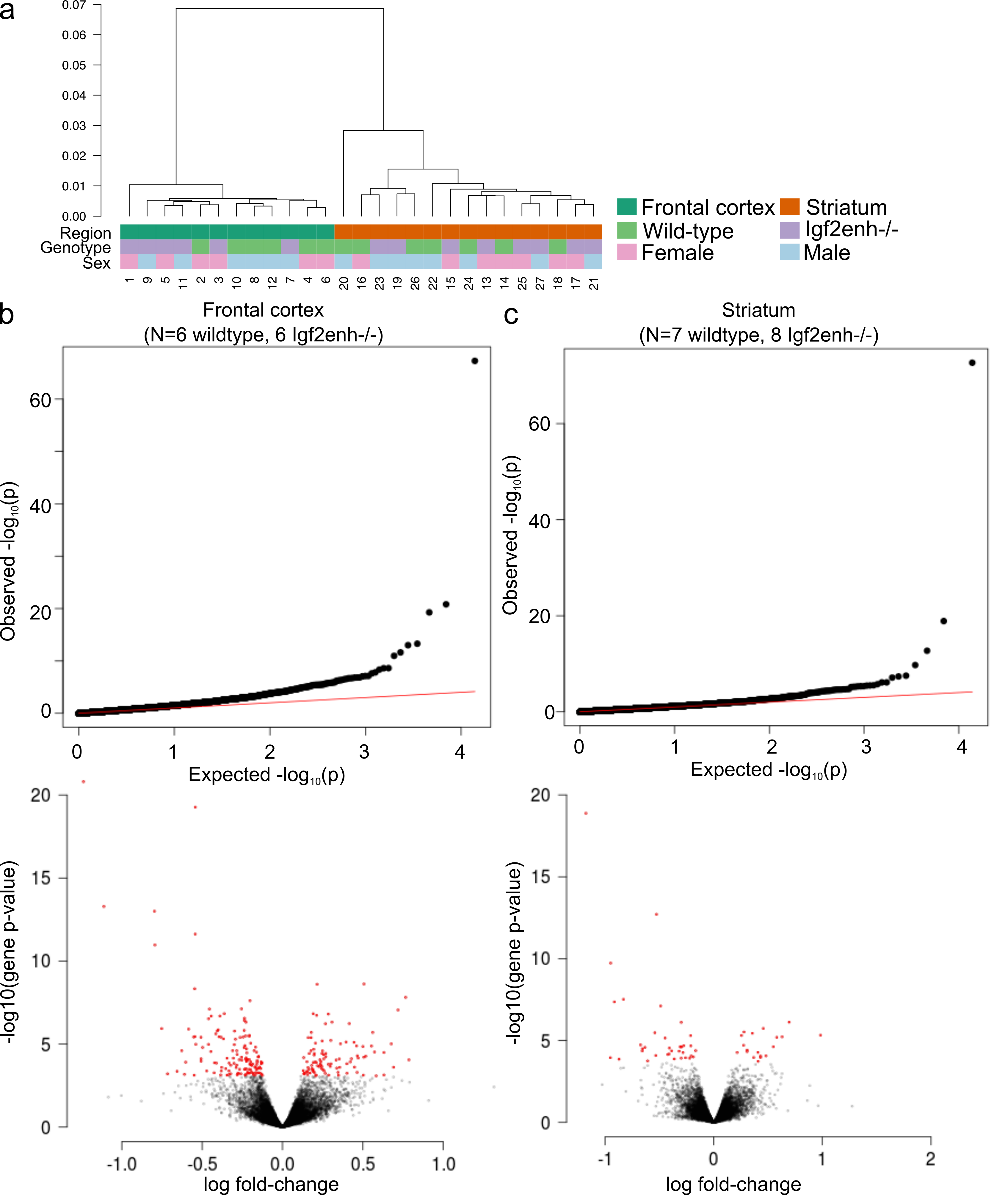


**Supplementary Figure 14.** Differential expression in frontal cortex and striatum of adult mice with an enhancer deletion at the *Igf2* locus. (**a**) Hierarchical clustering of normalized read counts (log counts per million (CPM), genes with CPM≥1 in all samples; n=13,490 genes; n=27 samples). Plot shows the clustering relationship with brain region, genotype, and sex. Clustering used pairwise (1-P), where P is sample Pearson correlation, and average linkage. (**b, c**) QQ-plots (top) and volcano plots (bottom) for gene-level differential expression in the frontal cortex (**b**) and striatum (**c**) of mice with an *Igf2* enhancer deletion relative to wild-type mice. In the volcano plots, each dot represents data for one gene; x-axis shows effect size and y-axis shows nominal gene-level significance; dots in red are genes with *q*<0.05. generalized linear regression in edgeR ^6^; Frontal cortex (**b**) n=6 wild-type and 6 *Igf2*enh-/- mice; n=13,490 genes tested, 232 with *q*<0.05 and striatum (**c**) n=7 wild-type, 8 *Igf2*enh-/- mice; n=14,016 genes tested, 56 with *q*<0.05. For clarity *Glo1* data point is omitted (logFC=-1.2, *p*<10^-37^).


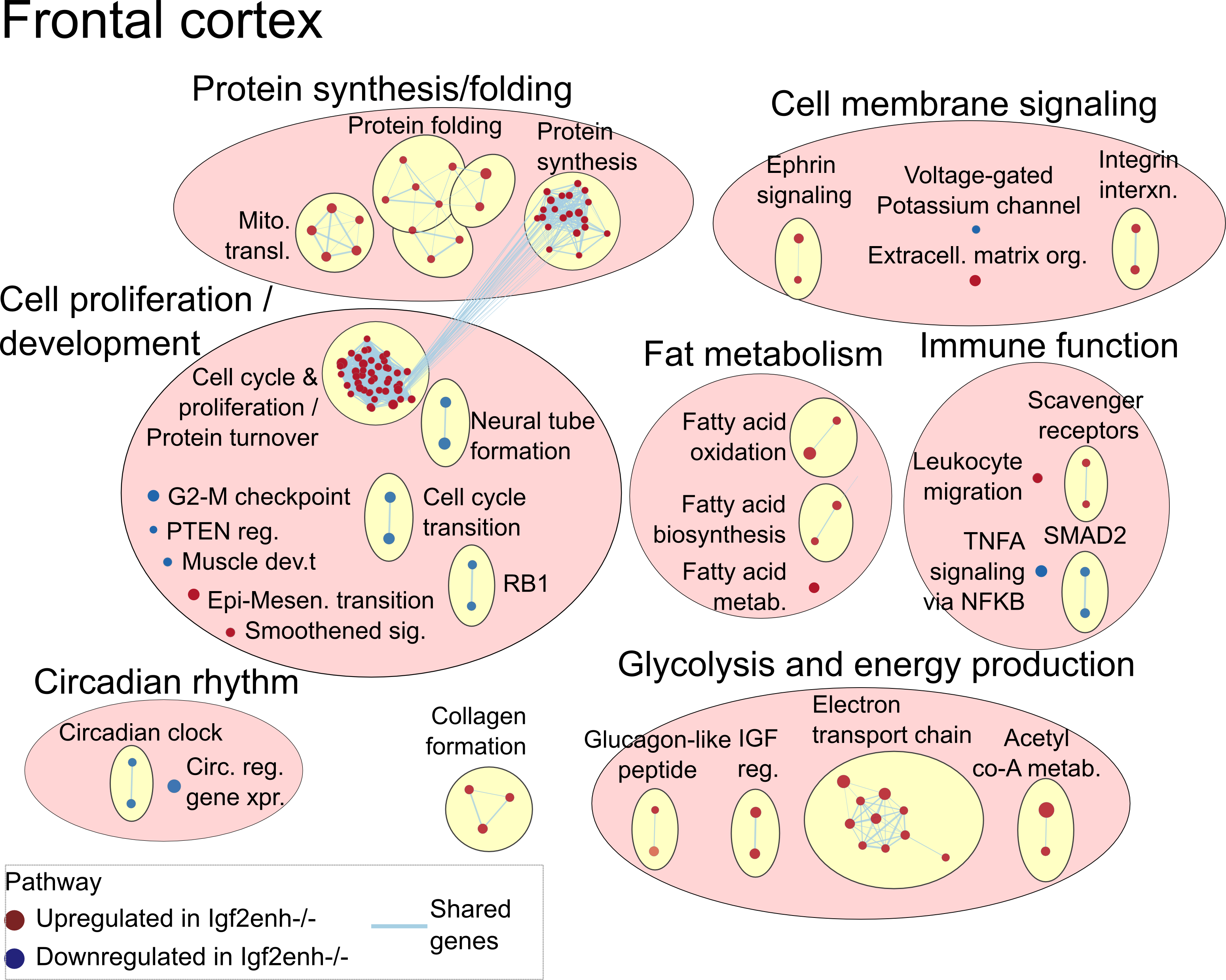


**Supplementary Figure 15.** Pathways enriched in transcriptomic alterations in the frontal cortex of *Igf2*enh-/- mice, relative to wildtype mice (n=6 wildtype, 6 *Igf2*enh-/- mice). Enrichment map where nodes are significant pathways (*q*<0.05; 143 of 6,321 pathways tested; pre-ranked GSEA^10^), with node fill indicating whether genes are up- (red) or down-regulated (blue) in the *Igf2*enh-/- mice. Edges indicate shared genes. Pathways were clustered using AutoAnnotate (yellow circle). Similarly-themed nodes were grouped (pink outer circles). Full set of pathways listed in Supplementary Data 14.

**
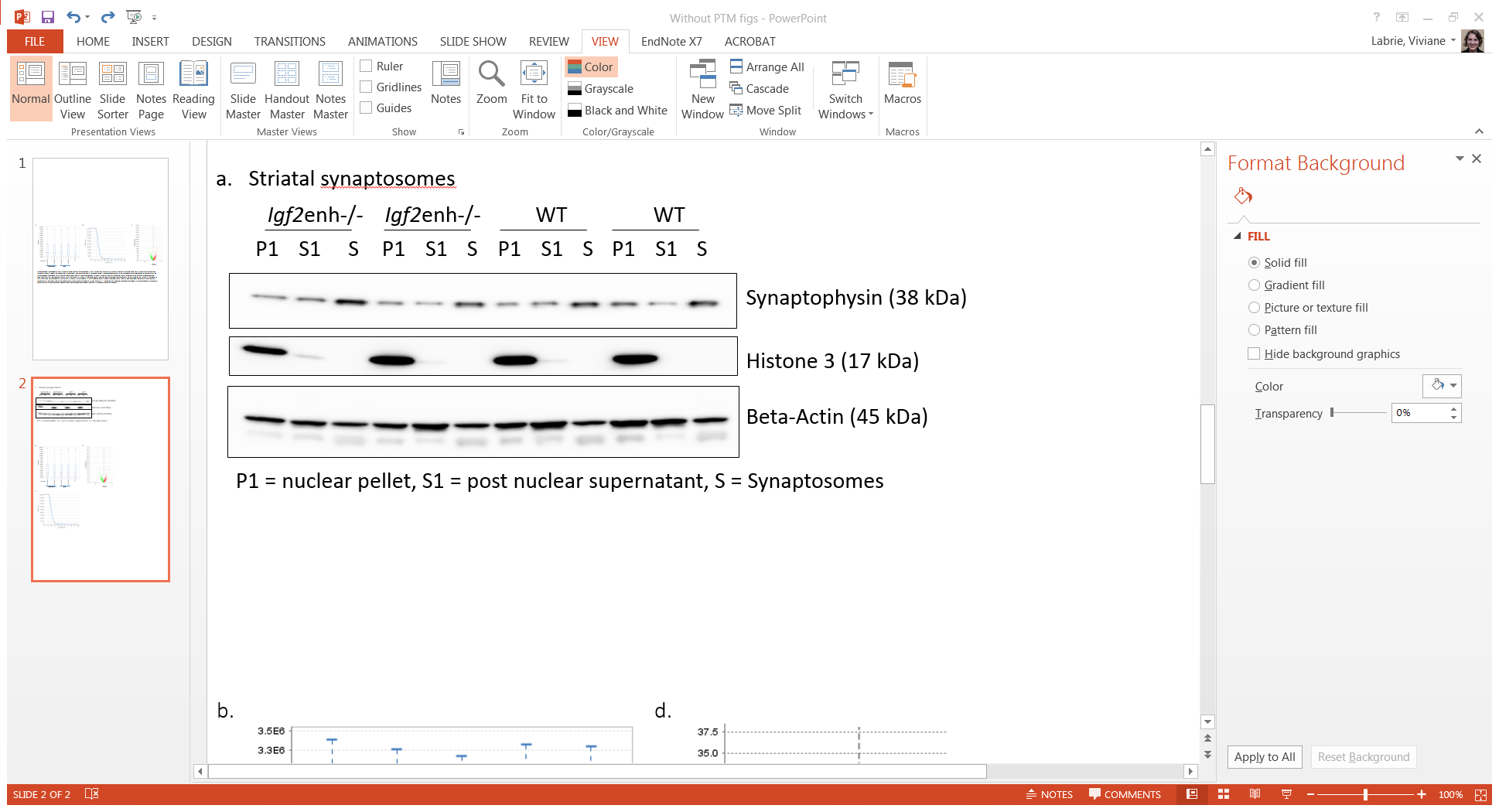
Supplementary Figure 16.** Synaptosomal proteome of the striatum of mice lacking the enhancer at *Igf2*. Mass spectrometry analysis of synaptic proteins isolated from the striatum of wild-type and *Igf2*enh-/- mice
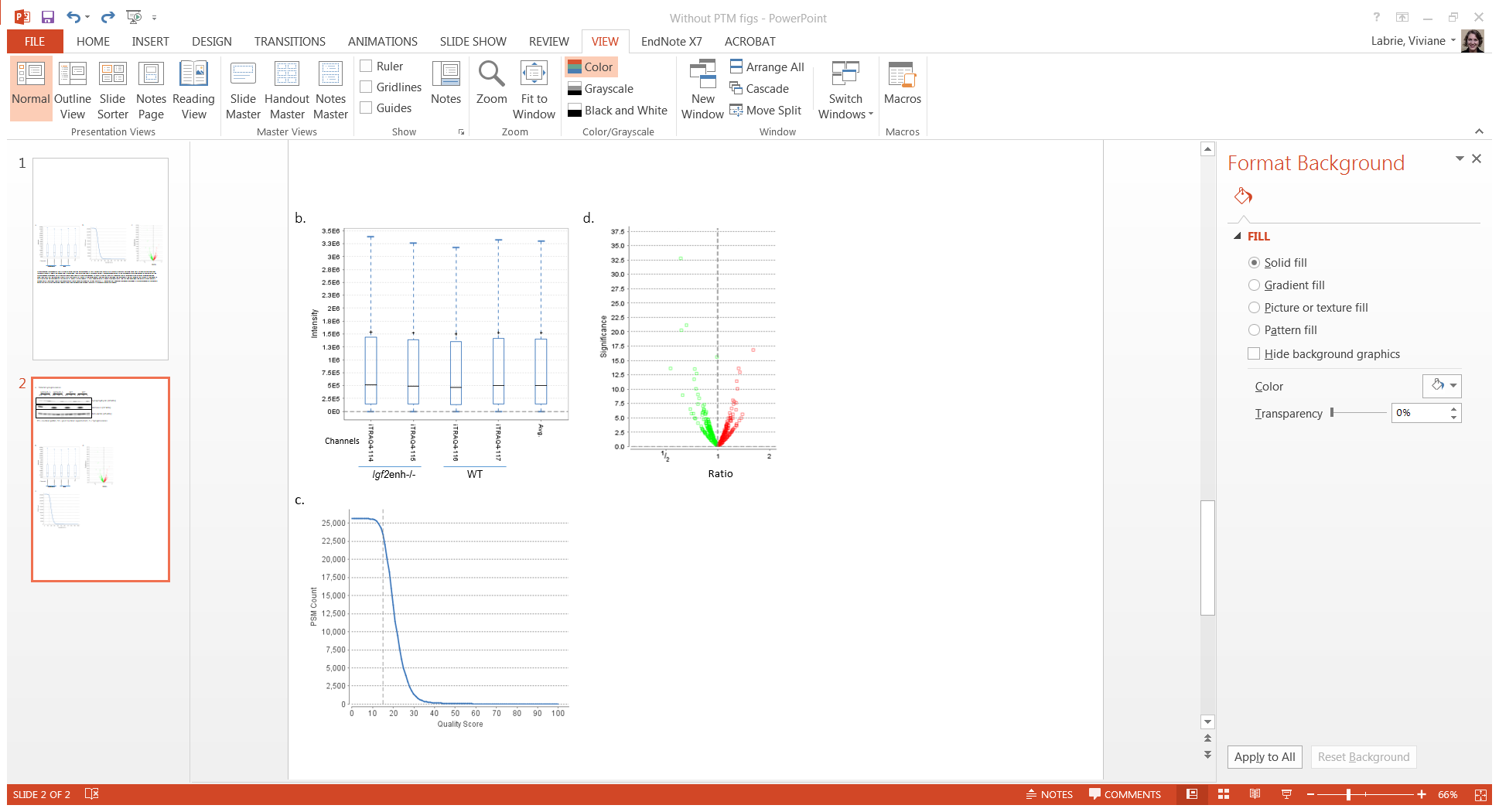
(2 pools per genotype, 3 mice/pool; n=6 wild-type and 6 *Igf2*enh-/- mice). iTRAQ-based quantitative proteomics was employed to characterize the synaptosomal proteome; 3619 proteins were identified with high confidence. (**a**) Immunoblot showing enrichment of synaptic proteins in the synaptosomal immunoprecipitated fraction. The synaptic protein synaptophysin is enriched and the nuclear protein histone H3 is absent in the synaptosomal preparation (β-actin is a loading control). (**b**) Quality control analysis showing similar representation of each iTRAQ-labelled pool. Box plots for the channel intensity distributions of all labels in the experiment. The horizontal dashed line represents the set reporter ion intensity threshold. A plot displaying the average distribution for all labels is also shown. (**c**) Plot showing quality score threshold (gray line) for peptide-spectrum match (PSM) counts. Higher quality peptides (which are more quantifiable) were retained for further analysis. (**d**) Volcano plot showing proteomic changes in synaptosomes of *Igf2*enh-/- mice relative to wild-type mice. Down- (red) and up-regulated (green) proteins in *Igf2*enh-/- mice are shown.

a.

**
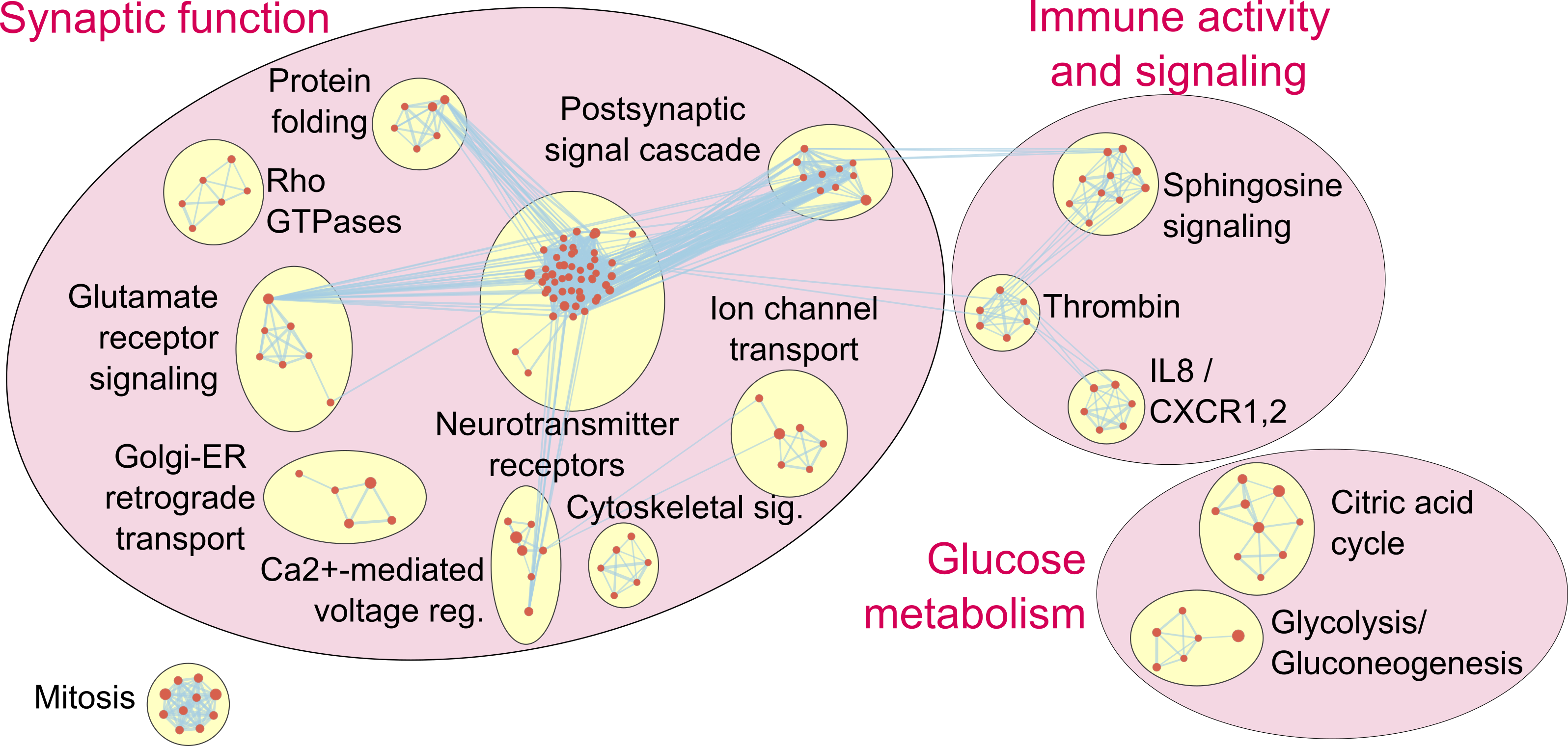
**

**Supplementary Figure 17.** Pathways enriched in proteins isolated from synaptosomes, relative to the rest of the mouse proteome. Image shows an enrichment map with nodes signifying significant pathways (hypergeometric test on 1,760 unique protein symbols corresponding to measured peptides, relative to proteins in all pathways (9,593 proteins). 320 pathways were significantly enriched (*q*<0.05). Pathways were visualized in Cytoscape using EnrichmentMap. AutoAnnotate was used to identify the top 15 clusters, shown here. Full set of pathways in Supplementary Data 19.

# Supplementary Tables

|  | **Controls** | **Schizophrenia** | **Bipolar** |
| --- | --- | --- | --- |
| **Num males** | 24 | 24 | 13 |
| **Num females** | 3 | 5 | 13 |
| **Age (mean+/-SD)** | 47.3 (9.56) | 48.0 (9.8) | 48.9 (14.2) |
| **Self-reported ethnicity** | 25 "White", 2 "Black" | 22 "White", 2 "Hispanic", 2 "Black", 3 unknown | All "White" |
| **Num CEU (genetically defined)** | 2 Female / 13 Male | 5 Female / 16 Male | 13 Female / 13 Male |
| **PMI** | 18.4 (5.9) | 19.4 (7) | 17.6 (7.2) |

**Supplementary Table 1.** Sample summary.

# Supplementary References

1. Xi, Y. & Li, W. BSMAP: whole genome bisulfite sequence MAPping program. *BMC bioinformatics* **10**, 232 (2009).

2. Quinlan, A.R. & Hall, I.M. BEDTools: a flexible suite of utilities for comparing genomic features. *Bioinformatics (Oxford, England)* **26**, 841-842 (2010).

3. Li, H.*, et al.* The Sequence Alignment/Map format and SAMtools. *Bioinformatics (Oxford, England)* **25**, 2078-2079 (2009).

4. Bolger, A.M., Lohse, M. & Usadel, B. Trimmomatic: a flexible trimmer for Illumina sequence data. *Bioinformatics (Oxford, England)* **30**, 2114-2120 (2014).

5. Li, H. Tabix: fast retrieval of sequence features from generic TAB-delimited files. *Bioinformatics (Oxford, England)* **27**, 718-719 (2011).

6. Robinson, M.D., McCarthy, D.J. & Smyth, G.K. edgeR: a Bioconductor package for differential expression analysis of digital gene expression data. *Bioinformatics (Oxford, England)* **26**, 139-140 (2010).

7. Dobin, A.*, et al.* STAR: ultrafast universal RNA-seq aligner. *Bioinformatics (Oxford, England)* **29**, 15-21 (2013).

8. Fortin, J.P., Triche, T.J., Jr. & Hansen, K.D. Preprocessing, normalization and integration of the Illumina HumanMethylationEPIC array with minfi. *Bioinformatics (Oxford, England)*  (2016).

9. Shannon, P.*, et al.* Cytoscape: a software environment for integrated models of biomolecular interaction networks. *Genome research* **13**, 2498-2504 (2003).

10. Subramanian, A.*, et al.* Gene set enrichment analysis: a knowledge-based approach for interpreting genome-wide expression profiles. *Proceedings of the National Academy of Sciences of the United States of America* **102**, 15545-15550 (2005).

11. Merico, D., Isserlin, R., Stueker, O., Emili, A. & Bader, G.D. Enrichment map: a network-based method for gene-set enrichment visualization and interpretation. *PloS one* **5**, e13984 (2010).

12. Kucera, M., Isserlin, R., Arkhangorodsky, A. & Bader, G.D. AutoAnnotate: A Cytoscape app for summarizing networks with semantic annotations. *F1000Research* **5**, 1717 (2016).

13. Newman, A.M.*, et al.* Robust enumeration of cell subsets from tissue expression profiles. *Nature methods* **12**, 453-457 (2015).

14. Purcell, S.*, et al.* PLINK: a tool set for whole-genome association and population-based linkage analyses. *American journal of human genetics* **81**, 559-575 (2007).

15. Das, S.*, et al.* Next-generation genotype imputation service and methods. *Nature genetics* **48**, 1284-1287 (2016).

16. Loh, P.R., Palamara, P.F. & Price, A.L. Fast and accurate long-range phasing in a UK Biobank cohort. *Nature genetics* **48**, 811-816 (2016).

17. Croft, D.*, et al.* The Reactome pathway knowledgebase. *Nucleic acids research* **42**, D472-477 (2014).

18. Fabregat, A.*, et al.* The Reactome pathway Knowledgebase. *Nucleic acids research* **44**, D481-487 (2016).

19. Kandasamy, K.*, et al.* NetPath: a public resource of curated signal transduction pathways. *Genome biology* **11**, R3 (2010).

20. Mi, H.*, et al.* The PANTHER database of protein families, subfamilies, functions and pathways. *Nucleic acids research* **33**, D284-288 (2005).

21. Romero, P.*, et al.* Computational prediction of human metabolic pathways from the complete human genome. *Genome biology* **6**, R2 (2005).

22. Schaefer, C.F.*, et al.* PID: the Pathway Interaction Database. *Nucleic acids research* **37**, D674-679 (2009).

23. Szklarczyk, D.*, et al.* The STRING database in 2017: quality-controlled protein-protein association networks, made broadly accessible. *Nucleic acids research* **45**, D362-d368 (2017).

24. Van der Auwera, G.A.*, et al.* From FastQ data to high confidence variant calls: the Genome Analysis Toolkit best practices pipeline. *Current protocols in bioinformatics* **43**, 11.10.11-33 (2013).

25. Chris, L.*, et al.* SciNet: Lessons Learned from Building a Power-efficient Top-20 System and Data Centre. *Journal of Physics: Conference Series* **256**, 012026 (2010).

26. van Iterson, M., van Zwet, E.W. & Heijmans, B.T. Controlling bias and inflation in epigenome- and transcriptome-wide association studies using the empirical null distribution. *Genome biology* **18**, 19 (2017).
