# Supplementary material for: Differential methylation of enhancer at IGF2 is associated with abnormal dopamine synthesis in major psychosis
Source: Nat Commun. 2019 May 3;10:2046. doi: 10.1038/s41467-019-09786-7 (PMC6499808; doi:10.1038/s41467-019-09786-7)
Supplement: Supplementary file 2 — Description of Additional Supplementary Files [file 41467_2019_9786_MOESM2_ESM.pdf]

Description of Supplementary Files

File Name: Supplementary Data 1.

Description: Patient information.

File Name: Supplementary Data 2.

Description: Comb-p results for DMR calls in EPIC microarrays.

File Name: Supplementary Data 3.

Description: Pathway analysis of EPIC array probes.

File Name: Supplementary Data 4.

Description: Summary statistics for RNA sequencing.

File Name: Supplementary Data 5.

Description: Differential expression results for case-control RNAseq data.

File Name: Supplementary Data 6.

Description: Pathway analysis for transcriptomic data from GSEA.

File Name: Supplementary Data 7.

Description: Results of cis meQTL analysis of DMRs from the EPIC arrays.

File Name: Supplementary Data 8.

Description: Results of eQTL analysis of EPIC DMRs with SCZ GWAS hits and credible SNPs.

File Name: Supplementary Data 9.

Description: Genotype-disease interaction of cis-SNPs in linkage equilibrium in IGF2 DMR region.

File Name: Supplementary Data 10.

Description: Summary statistics for processing pipeline of targeted bisulfite sequencing (SeqCapEPI).

File Name: Supplementary Data 11.

Description: RNA-seq of wild-type and Igf2enh-/- mice.

File Name: Supplementary Data 12.

Description: Differential expression in Igf2enh-/- mice in frontal cortex.

File Name: Supplementary Data 13.

Description: Differential expression in Igf2enh-/- mice in striatum.

File Name: Supplementary Data 14.

Description: Pathway enrichment results for Igf2enh-/- mice for frontal cortex.

File Name: Supplementary Data 15.

Description: Pathway enrichment results for Igf2enh-/- mice for striatum.

File Name: Supplementary Data 16.

Description: Enriched synaptosomal proteins in striatum of wild type mice, relative to Igf2enh-/- mice. Ratio values above 1 are higher in the wildtype; those under 1 are higher in Igf2enh-/- mice.

File Name: Supplementary Data 17.

Description: Mouse synaptosome wt vs Igf2enh-/- pathway analysis results from Metacore (Disease pathways).

File Name: Supplementary Data 18.

Description: Mouse synaptosome wt vs Igf2enh-/- pathway analysis results from Metacore (Biological processes).

File Name: Supplementary Data 19.

Description: Mouse synaptosome enrichment analysis (Biological processes).
