# Supplementary material for: Differential methylation of enhancer at IGF2 is associated with abnormal dopamine synthesis in major psychosis
Source: Nat Commun. 2019 May 3;10:2046. doi: 10.1038/s41467-019-09786-7 (PMC6499808; doi:10.1038/s41467-019-09786-7)
Supplement: Supplementary file 22 — Reporting Summary [file 41467_2019_9786_MOESM22_ESM.pdf]

nature research

Corresponding author(s): *Double-blind peer review submissions: write DBPR and your manuscript number here instead of author names.*

Reporting Summary

Nature Research wishes to improve the reproducibility of the work that we publish. This form provides structure for consistency and transparency in reporting. For further information on Nature Research policies, see Authors & Referees and the Editorial Policy Checklist.

Statistical parameters

When statistical analyses are reported, confirm that the following items are present in the relevant location (e.g. figure legend, table legend, main text, or Methods section).

n/a	Confirmed	
☐	☒	The exact sample size (*n*) for each experimental group/condition, given as a discrete number and unit of measurement
☐	☒	An indication of whether measurements were taken from distinct samples or whether the same sample was measured repeatedly
☐	☒	The statistical test(s) used AND whether they are one- or two-sided<br>*Only common tests should be described solely by name; describe more complex techniques in the Methods section.*
☐	☒	A description of all covariates tested
☐	☒	A description of any assumptions or corrections, such as tests of normality and adjustment for multiple comparisons
☐	☒	A full description of the statistics including central tendency (e.g. means) or other basic estimates (e.g. regression coefficient) AND variation (e.g. standard deviation) or associated estimates of uncertainty (e.g. confidence intervals)
☐	☒	For null hypothesis testing, the test statistic (e.g. *F*, *t*, *r*) with confidence intervals, effect sizes, degrees of freedom and *P* value noted<br>*Give P values as exact values whenever suitable.*
☒	☐	For Bayesian analysis, information on the choice of priors and Markov chain Monte Carlo settings
☐	☒	For hierarchical and complex designs, identification of the appropriate level for tests and full reporting of outcomes
☐	☒	Estimates of effect sizes (e.g. Cohen's *d*, Pearson's *r*), indicating how they were calculated
☐	☒	Clearly defined error bars<br>*State explicitly what error bars represent (e.g. SD, SE, CI)*

Our web collection on statistics for biologists may be useful.

Software and code

Policy information about availability of computer code

Data collection	Illumina GenomeStudio: Microarray intensities were scanned and quantified for EPIC DNA methylation microarrays and for genotyping microarrays<br>SeqCap EPI capture probes (Roche Sequencing Solutions): Probe design<br>PEAKS Studio 8.5: peptide identification of mass spectrometry data
Data analysis	R v3.3.1 and 3.4.0: Basic data analysis<br>BioConductor packages (BioBase v 2.36.2): Analysis on genomic intervals in methylation microarray, targeted bisulfite sequencing, genotyping data, and RNA sequencing<br>BSMap 2.74: Alignment of targeted bisullfite sequencing<br>Picard 2.9.4-SNAPSHOT: Preprocessing of bisulfite sequencing and genotype data<br>Bedtools 2.26: Preprocessing of bisulfite sequencing and genotype data<br>Samtools 1.5: Preprocessing of bisulfite sequencing and genotype data<br>Bamutils 1.0.14: Clip overhanging reads that distort methylation estimates in targeted bisulfite sequencing data.<br>FastQC v0.11.5: Quality control of raw sequencing reads for bisulfite sequencing and RNA sequencing data<br>Trimmomatic 0.36: Removal of adaptor sequences for targeted bisulfite sequencing data<br>Tabix (HTSutils) 1.5 : Indexing of bisulfite methylation calls for fast retrieval<br>edgeR 3.18.1: Differential expression analysis of RNA sequencing raw reads<br>Trimgalore 0.5.0: Trimming of RNA sequencing raw reads<br>STAR 2.5.3a: Alignment of RNA sequencing reads

Minfi 1.22.1: Preprocessing of methylation EPIC microarrays
Cytoscape 3.5.1: Visualization of pathway enrichment results as network diagrams
GSEA 3.0: Pathway analysis of gene expression data
EnrichmentMap 3.1.0RC4: Visualization of pathway enrichment results in network format
AutoAnnotate 1.2: Clustering and labelling of major themes in the EnrichmentMap used to visualize pathway enrichment
CIBERSORT: Perform cell-type deconvolution based on gene expression data
Plink 1.90b4.9: Quality control, filtering and reformatting of genotype data
Michigan Imputation Server: Server that runs complete imputation process of genotype microarray data
Eagle 2.3: Genomic imputation of genotype microarray data (used on the Michigan Imputation Server)
Check-Bim: Quality control of genotype data prior to submission to Michigan Imputation Server
String (https://string-db.org/): Find evidence for protein-protein interactions
MetaCore (https://clarivate.com/products/metacore/): Pathway analysis of mass spectrometry
GATK 3.8.0: Haplotype calls in RNA sequencing data for sample identity matching with genotype data
LiftOver (UCSC): Convert genomic coordinates to the same genome build across platforms, for cross platform analysis.

For manuscripts utilizing custom algorithms or software that are central to the research but not yet described in published literature, software must be made available to editors/reviewers upon request. We strongly encourage code deposition in a community repository (e.g. GitHub). See the Nature Research guidelines for submitting code & software for further information.

Data

Policy information about availability of data

All manuscripts must include a data availability statement. This statement should provide the following information, where applicable:

- Accession codes, unique identifiers, or web links for publicly available datasets
- A list of figures that have associated raw data
- A description of any restrictions on data availability

Raw and processed data for data generated in this work have been deposited at the Gene Expression Omnibus under the SuperSeries accession number GSE112525. These include subseries for human DNA methylation arrays (GSE112179), RNA-sequencing (GSE112523), bisulfite targeted sequencing (GSE112524), and genotyping arrays (GSE113093) , and transcriptome profiling of mouse brains (GSE120423). These data are associated with Fig. 1, 2, 4 and Supplementary Fig. 3-9 and 14-15.
The chromatin conformation analysis in human prefrontal cortex, as shown in Fig. 3a and Supplementary Fig. 10, used peaks provided from the 3D Interaction Database at https://www.kobic.kr/3div/.
Protein-protein interaction networks shown in Fig. 4c and Supplementary Fig. 7c were obtained from the STRING database (https://string-db.org/).
Software used to produce the results in this work is available at https://github.com/shraddhapai/EpigeneticsPsychosis/ and will be made publicly available upon publication.
Data for Fig. 3b-e and Supplementary Fig. 12 and 13 are available in the Source Data file.

Field-specific reporting

Please select the best fit for your research. If you are not sure, read the appropriate sections before making your selection.

☒ Life sciences ☐ Behavioural & social sciences ☐ Ecological, evolutionary & environmental sciences

For a reference copy of the document with all sections, see nature.com/authors/policies/ReportingSummary-flat.pdf

Life sciences study design

All studies must disclose on these points even when the disclosure is negative.

Sample size

DNA methylation status was measured at 812,663 CpG sites in neuronal cells sorted from post mortem frontal cortex samples of 28 controls and 55 cases (29 schizophrenia, 26 bipolar disorder) (100 samples including technical replicates). Genotypes were measured in the same samples using Infinium PsychArray-24 microarrays (228,369 SNPs). RNA-sequencing was used to measure the transcriptome in 17 controls and 17 cases (7 schizophrenia, 10 bipolar disorder); samples were a subset of those profiled for genome-wide DNA methylation and tissue was unsorted frontal cortex tissue. Targeted bisulfite sequencing was performed on neuronal DNA of 13 cases and 13 controls (total of 34 samples including technical replicates), and separately on non-neuronal DNA from 10 cases and 12 controls; samples were a subset of those profiled for genome-wide DNA methylation using microarrays and tissue was from the same sample of frontal cortex for which methylation microarrays, genotyping, and RNA-sequencing was performed.

Using RNA-sequencing, the transcriptome was profiled in wild-type 129S1 mice and those with genetic deletions of the target regulatory element, for both the frontal cortex (6 wildtype and 6 knock-out) and striatum (7 wildtype, 8 knockout). Mass spectrometry was used to profile the synaptosomal proteome in 6 wildtype and 6 knock out mice (2 pools per genotype, with 3 mice/pool).

Data exclusions

Methylation microarrays: One sample was excluded as it did not cluster with other neuronal DNA samples during data exploration; no other samples were excluded.
RNA sequencing: No samples were excluded.
Targeted bisulfite sequencing: Data exploration identified three samples that did not cluster with other samples; these three were excluded from further analysis.
Genotype arrays: One sample failed quality control tests and was excluded.

Replication	The main finding of the paper pertains to identification of a locus of differential DNA methylation in neurons of individuals with major psychosis, using methylation microarrays. We validated this finding by 1) repeating the genome-wide differential methylation analysis with samples with European genetic ancestry, 2) by limiting to European males, 3) by controlling for lifestyle variables, including smoking and antipsychotic use, and 4) using an alternate technique of targeted bisulfite sequencing.<br><br>We also found that methylation changes at our target site affected protein levels of Tyrosine hydroxylase, and replicated this finding using mouse brain samples.
Randomization	All samples were randomized in the study (in the isolation of neuronal nuclei and DNA methylation array, bisulfite padlock probe-seq, SNP-array, and RNA-seq library preparation).
Blinding	An experimenter blind to the sample key performed the isolation of neuronal nuclei, the DNA methylation library preparation and arrays, SNP arrays, and RNA-seq library preparation.

Reporting for specific materials, systems and methods

Materials & experimental systems

n/a	Involved in the study
☐	☒ Unique biological materials
☐	☒ Antibodies
☒	☐ Eukaryotic cell lines
☒	☐ Palaeontology
☐	☒ Animals and other organisms
☐	☒ Human research participants

Methods

n/a	Involved in the study
☒	☐ ChIP-seq
☐	☒ Flow cytometry
☒	☐ MRI-based neuroimaging

Unique biological materials

Policy information about availability of materials

Obtaining unique materials	Postmortem human brain tissue used in this study was obtained from the NIH Neurobiobank. This tissue bank can be contacted for this material. The study protocol was approved by the institutional review board at the Centre for Addiction and Mental Health and the Van Andel Research Institute (IRB #15025).

Antibodies

Antibodies used	anti-NeuN Alexa Fluor 488 antibody (Abcam), anti-synaptophysin antibody (Abcam), anti-histone 3 antibody (Abcam), anti-actin antibody (Millipore), anti-tyrosine hydroxylase antibody (PelleFreez Biologicals), anti-NeuN antibody (Cell Signaling), anti-internexin neuronal intermediate filament protein antibody (Sigma)
Validation	anti-NeuN Alexa Fluor 488 antibody [EPR12763]: Abcam (ab190195), manufacturer confirmed suitable for Flow Cytometry, immunohistochemistry, immunofluorescence. Host species: Rabbit, monoclonal. Reacts with: Human, Mouse, Rat. https://www.abcam.com/neun-antibody-epr12763-neuronal-marker-alexa-fluor-488-ab190195.html<br><br>anti-synaptophysin antibody [SY38]: Abcam (ab8049), manufacturer confirmed suitable for western blot, flow cytometry, immunofluorescence, immunohistochemistry. Host species: Mouse, monoclonal. Reacts with: Mouse, Rat, Hamster, Cow, Human. https://www.abcam.com/synaptophysin-antibody-sy38-ab8049.html<br><br>anti-histone 3 antibody: Abcam (ab1791), manufacturer confirmed suitable for western blot, flow cytometry, immunofluorescence, immunohistochemistry, ChIP, electron microscopy, immunoprecipitation. Host species: Rabbit, polyclonal. React with: Mouse, Rat, Chicken, Dog, Human, Saccharomyces cerevisiae, Xenopus laevis, Arabidopsis thaliana, Caenorhabditis elegans, Drosophila melanogaster, Ferret, Indian muntjac, Schizosaccharomyces pombe, Zebrafish, Silk worm, Dictyostelium discoideum, Rainbow trout, Trypanosoma cruzi, Neurospora crassa, Toxoplasma gondii, Rice, Schistosoma mansoni, Candida albicans, Cyanidioschyzon merolae. https://www.abcam.com/histone-h3-antibody-nuclear-loading-control-and-chip-grade-ab1791.html<br><br>anti-actin antibody, clone C4: Millipore (MAB1501, lot 2757213), manufacturer confirmed for western blot, immunohistochemistry, immunofluorescence, ELISA. Host species: Mouse, monoclonal. Reacts with: All animal species and cell types with actin. http://www.emdmillipore.com/US/en/product/Anti-Actin-Antibody-clone-C4,MM_NF-MAB1501<br><br>anti-tyrosine hydroxylase antibody: Pelfreez Biologicals (P40101-150), manufacturer confirmed for western blot, immunofluorescence, immunohistochemistry. Host species: Rabbit, polyclonal. Reacts with: All mammalian tyrosine hydroxylase. http://www.pelfreez-bio.com/wp-content/uploads/2014/07/74075-PDS-P40101-Tyrosine-Hydroxylase-Antibody-Rabbit-Rev-02.pdf

anti-NeuN antibody (D3S3I): Cell Signaling (12943), manufacturer confirmed suitable for western blot, immunoprecipitation, immunohistochemistry, immunofluorescence, ChIP, flow cytometry. Host species: Rabbit, monoclonal. Reacts with: Human, Mouse, Rat. https://www.cellsignal.com/products/primary-antibodies/neun-d3s3i-rabbit-mab/12943

anti-internexin neuronal intermediate filament protein antibody: Sigma (HPA008057), manufacturer confirmed suitable for western blot, immunofluorescence, immunohistochemistry. Host species: Rabbit, polyclonal. Reacts with: Human, Mouse, Rat. https://www.sigmaaldrich.com/catalog/product/sigma/hpa008057?lang=en&region=US

Animals and other organisms

Policy information about studies involving animals; ARRIVE guidelines recommended for reporting animal research

Laboratory animals	129S1 strain. Males and Females, approximately 2.5 months old. All animal procedures were approved by the Institutional Animal Care Committee of the Van Andel Research Institute and complied with the requirements of the Institutional Animal Care and Use Committee (AUP # PIL-17-10-010).
Wild animals	Not applicable
Field-collected samples	Not applicable

Human research participants

Policy information about studies involving human research participants

Population characteristics	Information for all human frontal cortex samples is provided in Table S1 and Sample Summary in the Supplementary Tables.
Recruitment	Postmortem brain tissue samples obtained from the NIH NeuroBioBank. No individual was recruited specifically for this study.

Flow Cytometry

Plots

Confirm that:

☒ The axis labels state the marker and fluorochrome used (e.g. CD4-FITC).

☒ The axis scales are clearly visible. Include numbers along axes only for bottom left plot of group (a 'group' is an analysis of identical markers).

☒ All plots are contour plots with outliers or pseudocolor plots.

☒ A numerical value for number of cells or percentage (with statistics) is provided.

Methodology

Sample preparation	Neuronal nuclei were separated using a flow cytometry-based approach, similar to as previously described2, 3. Human brain tissue (250 mg) for each sample was minced in 2 mL PBSTA (0.3 M sucrose, 1X phosphate buffered saline (PBS), 0.1% Triton X-100). Samples were then homogenized in PreCellys CKMix tubes with a Minilys (Bertin Instruments) set at 3,000 rpm for three 5 sec intervals, 5 min on ice between intervals. Samples homogenates were filtered through Miracloth (EMD Millipore), followed by a rinse with an additional 2 mL of PBSTA. Samples were then placed on a sucrose cushion (1.4 M sucrose) and nuclei were pelleted by centrifugation at 4,000 × g for 30 min 4°C using a swinging bucket rotor. For each sample, the supernatant was removed and the pellet was incubated in 700 µl of 1X PBS on ice for 20 min. The nuclei were then gently resuspended and blocking mix (100 µl of 1X PBS with 0.5% BSA (Thermo Fisher Scientific) and 10% normal goat serum (Gibco) was added to each sample. NeuN-488 (1:500; Abcam) was added and samples were incubated 45 min at 4°C with gentle mixing. Immediately prior to flow cytometry sorting, nuclei were stained with 7-AAD (Thermo Fisher Scientific) and passed through a 30 µM filter (SystemX). Nuclei positive for 7-AAD and either NeuN+ (neuronal) or NeuN- (non-neuronal) were sorted using an Influx (BD Biosciences) at the Faculty of Medicine Flow Cytometry Facility at the University of Toronto (Toronto, ON, Canada). Approximately 1 million NeuN+ nuclei were sorted for each sample. Immediately, after sorting nuclei were placed on ice and then precipitated by raising the volume to 10 mL with 1X PBS and adding 2 mL 1.8 M sucrose, 50 µl 1M $CaCl_2$ and 30 µl $Mg(Ace)_2$ and centrifugation at 1,786 × g for 15 min at 4°C. The supernatant was removed from NeuN+ and NeuN- samples and pellets were stored at -80°C.
Instrument	Nuclei positive for 7-AAD and either NeuN+ (neuronal) or NeuN- (non-neuronal) were sorted using an Influx (BD Biosciences) at the Faculty of Medicine Flow Cytometry Facility at the University of Toronto (Toronto, ON, Canada).
Software	BD FACS Sorftware sorter software
Cell population abundance	Approximately 1 million NeuN+ nuclei were sorted for each brain tissue sample. Purity was determined by reanalysis of aliquots of NeuN+ and NeuN- cells, and purity was confirmed to be on average 96%.

Gating strategy

Nuclei positive for 7-AAD and either NeuN+ (neuronal) or NeuN– (non-neuronal) were sorted using an Influx (BD Biosciences) at the Faculty of Medicine Flow Cytometry Facility at the University of Toronto (Toronto, ON, Canada). Gating was based on unstained, NeuN+ only, and 7-AAD only controls (each independently run to determine the gating with FSC).

☒ Tick this box to confirm that a figure exemplifying the gating strategy is provided in the Supplementary Information.
